# Fighting Ebola with novel spore decontamination technologies for the military

**DOI:** 10.3389/fmicb.2015.00663

**Published:** 2015-08-12

**Authors:** Christopher J. Doona, Florence E. Feeherry, Kenneth Kustin, Gene G. Olinger, Peter Setlow, Alexander J. Malkin, Terrance Leighton

**Affiliations:** ^1^U.S. Army Natick – Soldier RD&E Center, Warfighter Directorate, Natick, MAUSA; ^2^Department of Chemistry, Emeritus, Brandeis University, Waltham, MAUSA; ^3^National Institute of Allergy and Infectious Diseases, Integrated Research Facility – Division of Clinical Research, Fort Detrick, MDUSA; ^4^Department of Molecular Biology and Biophysics, University of Connecticut Health Center, Farmington, CTUSA; ^5^Biosciences and Biotechnology Division, Physical and Life Sciences Directorate, Lawrence Livermore National Laboratory, Livermore, CAUSA; ^6^Children’s Hospital – Oakland Research Institute, University of California San Francisco - Benioff, Oakland, CAUSA

**Keywords:** Ebola, decontamination technologies, spores, chrloine dioxide, military medicine

## Abstract

Recently, global public health organizations such as Doctors without Borders (MSF), the World Health Organization (WHO), Public Health Canada, National Institutes of Health (NIH), and the U.S. government developed and deployed Field Decontamination Kits (FDKs), a novel, lightweight, compact, reusable decontamination technology to sterilize Ebola-contaminated medical devices at remote clinical sites lacking infra-structure in crisis-stricken regions of West Africa (medical waste materials are placed in bags and burned). The basis for effectuating sterilization with FDKs is chlorine dioxide (ClO_2_) produced from a patented invention developed by researchers at the US Army Natick Soldier RD&E Center (NSRDEC) and commercialized as a dry mixed-chemical for bacterial spore decontamination. In fact, the NSRDEC research scientists developed an ensemble of ClO_2_ technologies designed for different applications in decontaminating fresh produce; food contact and handling surfaces; personal protective equipment; textiles used in clothing, uniforms, tents, and shelters; graywater recycling; airplanes; surgical instruments; and hard surfaces in latrines, laundries, and deployable medical facilities. These examples demonstrate the far-reaching impact, adaptability, and versatility of these innovative technologies. We present herein the unique attributes of NSRDEC’s novel decontamination technologies and a Case Study of the development of FDKs that were deployed in West Africa by international public health organizations to sterilize Ebola-contaminated medical equipment. FDKs use bacterial spores as indicators of sterility. We review the properties and structures of spores and the mechanisms of bacterial spore inactivation by ClO_2_. We also review mechanisms of bacterial spore inactivation by novel, emerging, and established non-thermal technologies for food preservation, such as high pressure processing, irradiation, cold plasma, and chemical sanitizers, using an array of *Bacillus subtilis* mutants to probe mechanisms of spore germination and inactivation. We employ techniques of high-resolution atomic force microscopy and phase contrast microscopy to examine the effects of γ-irradiation on bacterial spores of *Bacillus anthracis*, *Bacillus thuringiensis*, and *Bacillus atrophaeus* spp. and of ClO_2_ on *B. subtilis* spores, and present in detail assays using spore bio-indicators to ensure sterility when decontaminating with ClO_2_.

## 1. Introduction

Innovation in Science and Technology comes from myriad sources, such as thinking outside-the-box, applying expertise to new areas, or adapting novel technologies that advance the frontiers of knowledge to fill needs in the commercial marketplace for consumers or to meet critical capability gaps on the battlefield. Researchers at the U.S. Army Natick Soldier Research, Development and Engineering Center (NSRDEC) have invented and patented (Doona et al., 2014) an ensemble of novel decontamination technologies (**Table 1**) involving innovative dry, mixed-chemical technologies designed to be lightweight, compact, portable, easy-to-carry, energy-independent, flameless, almost waterless, inexpensive, safe to end-users and the environment (“green” technologies), and effective in addressing a diverse array of decontamination applications in far-forward military deployments or other high-intensity, austere environments by using the disinfectant chlorine dioxide (ClO_2_). These characteristics also make NSRDEC’s novel decontamination technologies well-suited for use during large-scale emergencies, natural disasters (Hurricane Katrina, tsunamis, superstorm Sandy) or in humanitarian relief in third-world countries.

**Table 1:** Acronyms and attributes of NSRDEC decontamination technologies.

Acronym	Technology	Attributes
NCC	**N**ovel **C**hemical **C**ombination	Dry powders mix with water
PCS	**P**ortable **C**hemical **S**terilizer	Plastic suitcase sterilizer
D-FENS	**D**isinfectant**-**sprayer **F**or **EN**vironmentally friendly **S**anitation	Collapsible handheld sprayer
D-FEND ALL	**D**isinfectant **F**or **EN**vironmentally friendly **D**econtamination, **ALL**-purpose	All purpose decontamination
CoD	**C**ompartment **o**f **D**efense	In-package disinfectant
FDK	**F**ield **D**econtamination **K**it	Ebola disinfectant


The use of chlorine dioxide (ClO_2_) to decontaminate *Bacillus anthracis* spores (causative agent of ‘Anthrax’) following the letter attacks on Washington, DC and other locations was facilitated by data from the author’s laboratories and other studies. These attacks were unprecedented in their use of mail processing delivery systems to create large-scale and wide-area *B. anthracis* spore contamination. They highlighted the need for more efficacious, agile and adaptive decontamination modalities that could extinguish primary and secondary nosocomial contact and transmission hazards. Concerns regarding transmission control of existential nosocomial diseases were further highlighted by the SARS and H1N1 pan epidemics. As Joshua Lederberg presciently observed in 1998 (*profiles.nlm.nih.gov/ps/access/BBBDLP.pdf*), with modern transportation and distribution infrastructure no infectious disease is more than 24 away from any location on the earth. Dr. Lederberg’s insight became salient with the emergence of the Ebola crisis emanating from West Africa and the concomitant challenges of controlling secondary chains of transmission at their origin and globally. In the summer of 2014, global public health and medical personnel adapted NSRDEC’s novel decontamination technologies for field use to fight the spread of Ebola by decontaminating medical equipment at remote clinical sites in West Africa.

Ebola virus disease (EVD) is a severe and often fatal disease in humans that is communicated between humans through contact with infected blood, organs or tissues, bodily fluids (saliva, sweat, vomit, urine, semen, and breast milk), or items they contaminate (clothing, bedding, gauze, needles and syringes, and medical equipment). In March, 2015, WHO estimated 24,842 cases and 10,299 deaths from this outbreak (World Health Organization [WHO], 2015a), and concerns of EVD heightened as EVD cases spread internationally. As an enveloped virus – one with a lipid and protein membrane – Ebola is vulnerable to chemical disinfectants, such as household bleach (OCl^-^) and chlorine dioxide (ClO_2_), which can be used to sanitize infected surfaces, patient rooms, and to sterilize contaminated medical equipment at remote clinical sites in West Africa (World Health Organization [WHO], 2014). In parts of the world that consume non-traditional foods (bats, monkeys, bush meat) as protein sources, basic food hygiene for preventing the transmission of biological hazards apply equally well to the Ebola virus (Anelich and Moy, 2014; World Health Organization [WHO], 2015b): Keep clean, Separate raw and cooked, Cook thoroughly (specifically, boiling for 5 min or heating for 60 min at *T* = 60°C inactivates the Ebola virus – Anelich and Moy, 2014), Keep food at safe temperatures, and Use safe water and raw materials.

Natick Soldier RD&E Center’s ensemble of patented novel decontamination technologies have the acronyms NCC, PCS, D-FENS, D-FEND ALL, and CoD (**Table 1**) and feature a variety of embodiments designed to produce ClO_2_ for killing bacterial spores (*B. anthracis*), vegetative pathogens (*Listeria monocytogenes*, *Escherichia coli*, etc.), viruses (Ebola), and bacteriophage in cross-cutting applications (Setlow et al., 2009), such as sterilizing surgical instruments, decontaminating textiles (uniforms, tents, shelters), sanitizing fresh fruits and vegetables procured in host nations, disinfecting wastewater, providing potable water quality and safety, and promoting hygiene by decontaminating surfaces bathrooms, showers, laundries, Army Field Kitchens, Navy Galleys, and deployable medical units. Deployments of military personnel worldwide generate thousands of tons of wastewater and food waste annually that support disease vectors capable of adversely affecting human health and account for Disease and Non-Battle Injuries (DNBIs) that at an average rate of 1.5% of assigned personnel, would have cost all branches of the military an estimated $32.5M annually (300 personnel per day) during the time of the Balkan conflicts in the 1990s! Finding inexpensive, convenient, and effective decontamination technologies improves hygiene and reduces incidences of DNBIs and other foodborne illnesses, thereby saving all branches of the military millions of dollars in medical costs and promoting health and well-being. For innovative patents such as these, Thomson Reuters in 2012 named the U.S. Army among the Top 100 Global Innovators (Foran, 2013 - available at http://www.army.mil/article/99816/).

Other technologies that decontaminate with gaseous ClO_2_ include electrically powered equipment to decontaminate facemasks worn as personal protective equipment (PPE) by emergency first-responders (Stubblefield and Newsome, 2015), and now Field Decontamination Kits (FDKs). FDKs are adapted from the commercial-off-the-shelf (COTS) version of NRDEC’s decontamination technologies and are presently being used by global public health organizations [Doctors without Borders (MSF), World Health Organization (WHO), Public Health Canada, National Institutes of Health (NIH)] and the U.S. government to sterilize Ebola-contaminated medical equipment at remote clinical sites in West Africa. While the Ebola virus is classified as Biosafety level 4 (BSL-4) due to the severity of disease in humans, the Ebola virus itself is relatively fragile and presently without a standard test assay under representative conditions even in a high-level containment facility. Bacterial spores therefore provide the standard test assay for sterility and/or decontamination efficacy, primarily because bacterial spores exhibit more resistance to chemical and physical decontamination methods. The author’s laboratories have studied the processes of *Bacillus* sporulation, spore germination, spore resistance and persistence, spore decontamination and spore structural biology for many years. Accordingly, we review bacterial spore properties, structures, and resistance mechanisms and focus on the mechanisms through which ClO_2_ inactivates bacterial spores as the indicators of efficient bio-decontamination.

**FIGURE 1:**
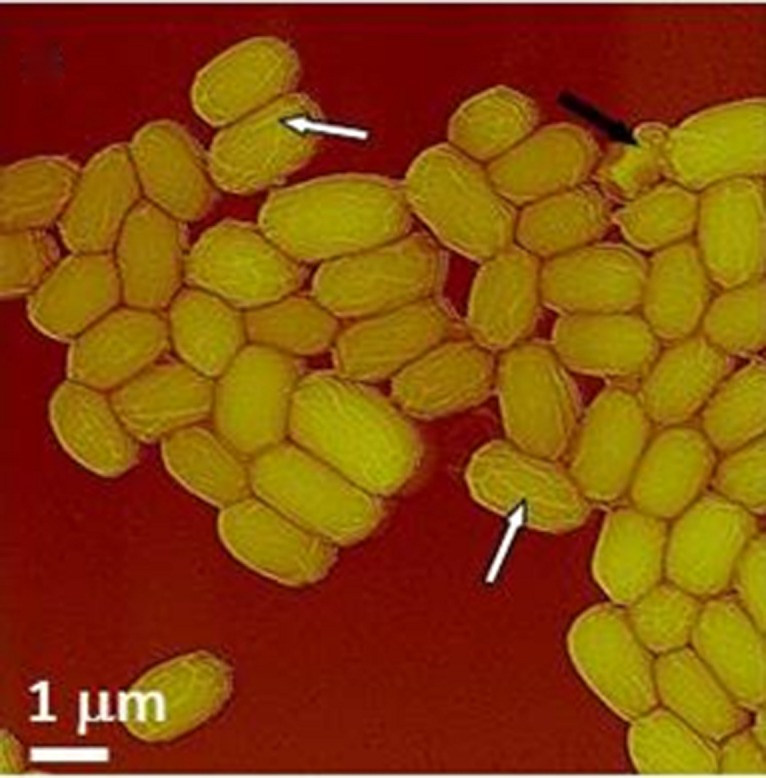
**AFM height image of native air-dried anthracis Sterne spores showing ridges (white arrows) and a collapsed spore (black arrow).** Further details are discussed with **Figure 9**.

We present the ontogeny of NSRDEC’s novel ClO_2_ decontamination technologies for spores that evolved to field-ready FDKs to meet an urgent need in protecting healthcare workers in West Africa from the spread of EVD during the heights of this international public health crisis. And just as ClO_2_ is also a non-thermal food processing technology for sanitizing fresh fruits and vegetables, we explore the characteristics of other Non-thermal technologies (chemical sanitizers, high pressure, irradiation, heat, plasmas, and UV light), particularly high pressure and γ-irradiation, that have also been used in the decontamination of *B. anthracis* (Cléry-Barraud et al., 2004) or other types of spores, and we also consider the mechanisms of bacterial spore inactivation by these agents. The ability of ClO_2_ to kill spores used as bio-indicators of sterility is also examined in detail, and we use high-resolution Atomic Force Microscopy (AFM) and phase contrast microscopy to examine the effects of nonthermal technologies on bacterial spores (**Figure 1**).

### 1.1. Additional Reference Materials

More information relating to this manuscript is available through the following links:

a. http://www.necn.com/news/new-england/Mass-Researchers-Create-Disinfectant-to-Fight-Ebola-280284792.html
b. http://www.metrowestdailynews.com/article/20141029/NEWS/141025763/0/SEARCH
  http://www.wcvb.com/news/natick-labs-innovation-could-help-prevent-spread-of-ebola/29370460?utm_source=hootsuite&utm_medium=facebook&utm_campaign=wcvb%2Bchannel%2B5%2Bboston
c. http://www.nbcnews.com/watch/nbc-news-channel/researchers-develop-ebola-disinfectant-348828227663
d. http://www.army.mil/article/136641/Natick_plays_key_role_in_helping_to_fight_spread_of_Ebola/
e. http://www.bizjournals.com/boston/blog/techflash/2015/04/why-a-u-s-army-research-facility-in-natick-is.html

## 2. Materials and Methods

### 2.1. Novel Redox Chemistry for the Production of ClO_2_

Natick Soldier RD&E Center’s bacterial spore decontamination technologies comprise a number of different dry chemical oxidation–reduction systems that mix with water in a glass beaker or plastic vessel to produce chlorine dioxide (ClO_2_) inside a carryable plastic suitcase, a rigid or a flexible plastic, hand-triggered spray-bottle, a sealable Mylar bag, or inside closable flexible or rigid plastic packaging (such as the plastic clamshell packaging used commercially to store and distribute fresh berries and other fresh or fresh-cut produce).

The NCC (**Table 1**) is the primary source of generating ClO_2_ and involves the oxidation–reduction reaction of chlorite (Sodium chlorite, Sigma-Aldrich Cat. No. 244155) and sulfite (Sodium sulfite, Sigma-Aldrich Cat. No. 239312) through the use of a unique chemical effector (Sodium ascorbate, Sigma-Aldrich A7631 – see Curtin et al., 2004) that initiates and controls the rate of the otherwise kinetically inert reaction, such that the production of ClO_2_ takes place at near-neutral pH on a practical and relatively short timescale (Curtin et al., 2014). The NCC improves the generation of ClO_2_ compared to existing methods, which include (i) the reduction of chlorate [ClO_3_^-^, Cl(V)] in high acid (HCl or H_2_SO_4_); (ii) the oxidation of chlorite [ClO_2_^-^, Cl(III)] by dichlorine [Cl_2_, Cl(0)], hypochlorite [OCl^-^, Cl(I)], or persulfate [S_2_O_8_^2-^, S(VII)], or (iii) the acidification of chlorite for the formation and subsequent disproportionation of chlorous acid [HClO_2_, Cl(III); Horváth et al., 2003]. While the PCS uses the NCC system to generate gaseous ClO_2_, D-FENS uses the NCC in conjunction with a novel two-step mixing process (i.e., *pre-concentration* followed by *ii. post-reaction dilution*), to generate aqueous ClO_2_ inside a rigid or collapsible plastic spray-bottle. The chemical systems for D-FEND ALL and CoD eliminate the need for the effector and involve only two chemical components and one-step mixing for greater user convenience, but neither has been formally disclosed yet through patent procedures.

### 2.2. NSRDEC’s Novel Decontamination Technologies

As reported previously (Doona et al., 2014) the novel decontamination technologies (NCC, PCS, D-FENS, D-FEND ALL, FDKs, CoD) were validated using laboratory chemical reagents and suitable challenge organisms and substrates to confirm sterility and material compatibility.

#### 2.2.1. The PCS

**FIGURE 2:**
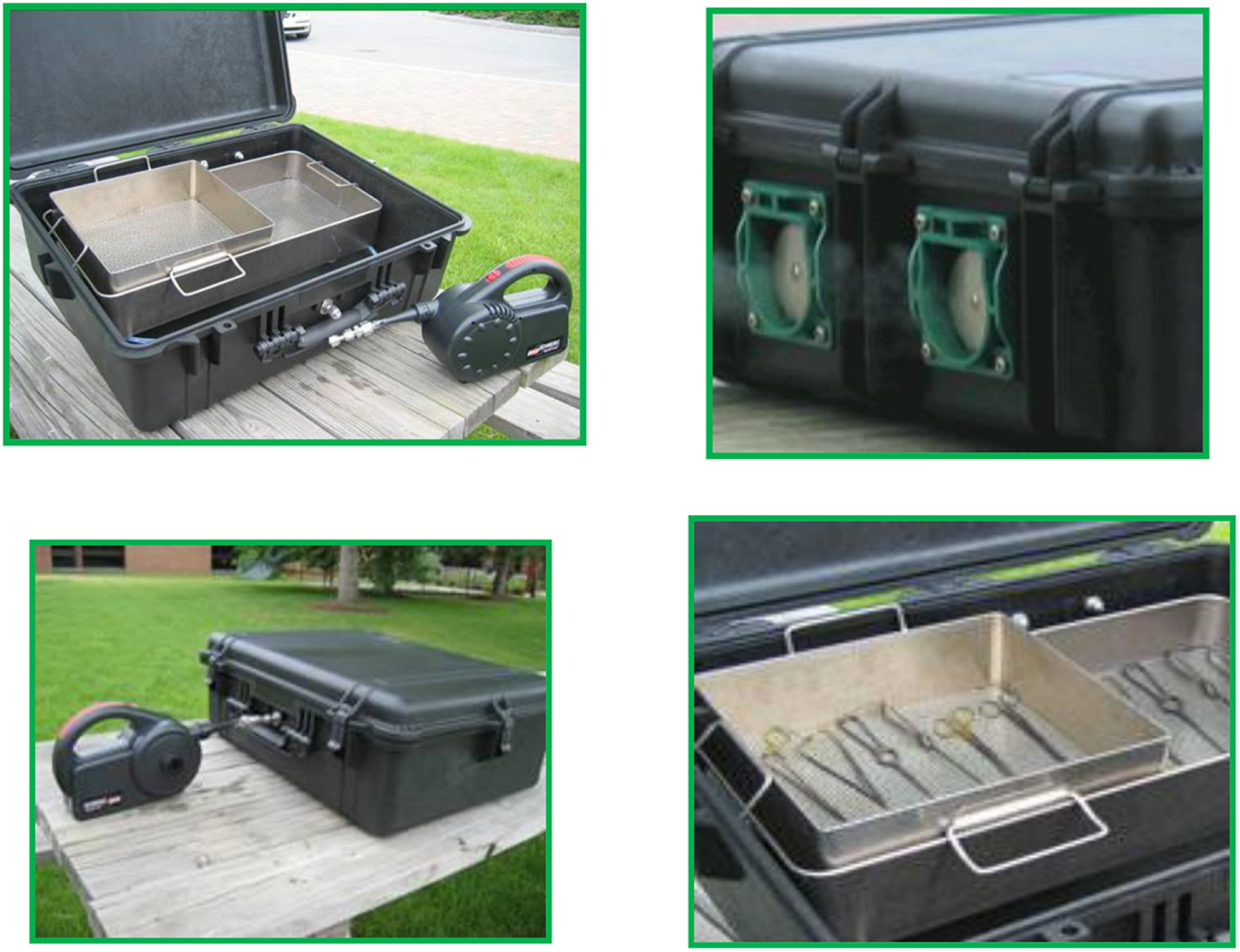
**The PCS (top left) consists of a rigid plastic suitcase embellished with reactors, valves (top right), scrubbers, and other design features (bottom left) to effectuate sterilization (bottom right) with ClO_2_ while protecting users and the environment**.

The PCS (**Figure 2**) is a Modern Field Autoclave, a revolutionary medical device invented to meet a stated Army need and an urgent battlefield demand for a field-portable, non-steam sterilizer technology that can be used by far-forward surgical teams (Doona et al., 2014). The PCS produces gaseous ClO_2_ and proceeds where no commercial device existed previously, with a 100% reduction in power usage, 98% reduction in water, 95% reduction in weight, and 96% reduction in cubic footprint compared to conventional steam autoclaves. The PCS used the NCC to sterilize live cultures of *Geobacillus stearothermophilus* spores in aqueous suspensions (recovered on Antibiotic Assay Medium with 1% soluble starch – see Feeherry et al., 1987; Doona et al., 2014), bio-indicators of *G. stearothermophilus* (BT Sure biological indicator (BI), Thermo Fisher Scientific Cat No. AY759X3) and *Bacillus atrophaeus* [EZ Test (EtO), SGM Biotech Inc., Cat. No. EZG/6] spores, live cultures of *L. monocytogenes* (recovered on Tryptic Soy Agar-Yeast Extract (TSAYE) incubated at *T* = 35°C for 48–96 h) and *E. coli* (recovered on Nutrient Agar) inoculated on hard surfaces (glass or stainless steel coupons) or on fresh whole tomatoes.

#### 2.2.2. D-FENS

**FIGURE 3:**
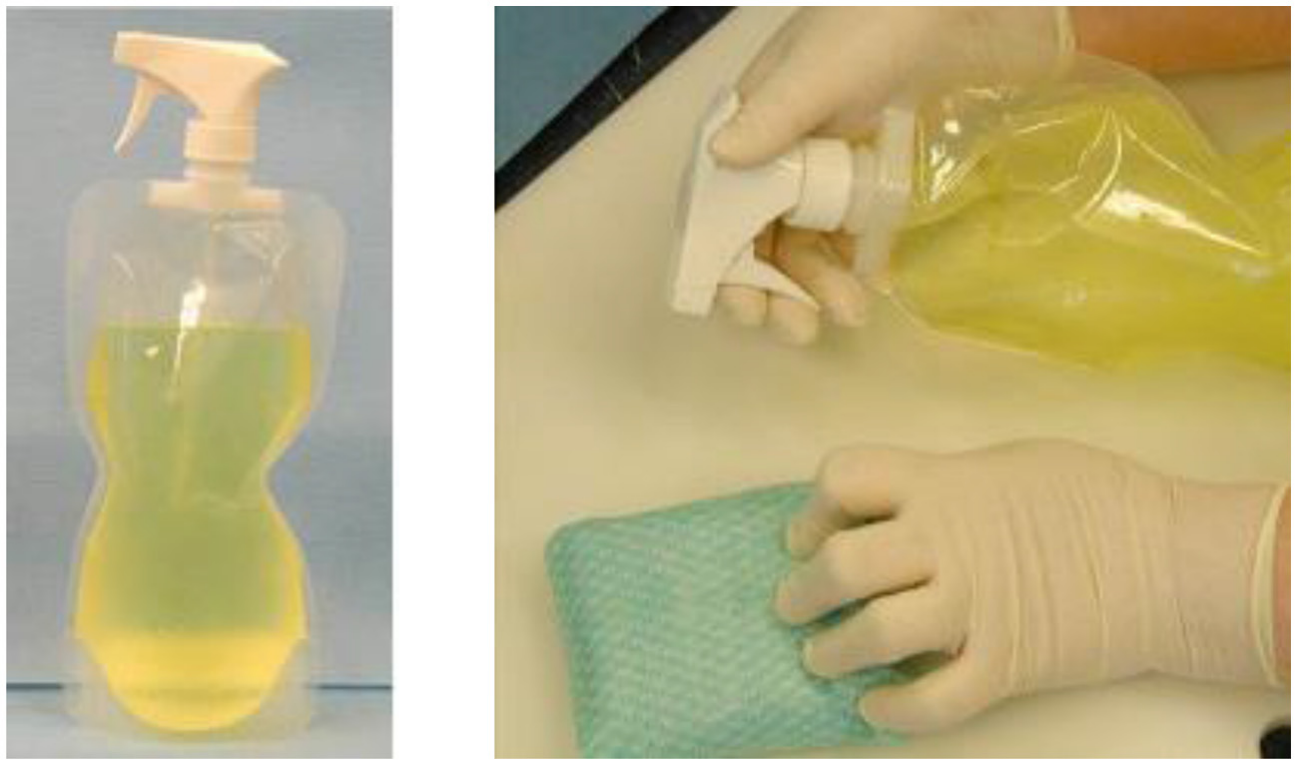
**The D-FENS sprayer generates aqueous ClO_2_ on-site and at point-of-use in a collapsible spray-bottle (**left**) and easily sprays ClO_2_ onto contact surfaces (**right**) to wipe away contaminating pathogens in Army Field Kitchens and Navy galleys**.

D-FENS (**Figure 3**) generates aqueous chlorine dioxide in a collapsible handheld spray-bottle (Doona et al., 2014) for decontaminating surfaces (fresh produce or medical, food handling, and surfaces in showers and latrines) anywhere large numbers of deployed personnel co-exist in close proximity. The D-FENS system uses the NCC with novel two-step mixing to generate aqueous ClO_2_ inside a rigid or collapsible plastic spray-bottle and was validated against a cocktail of *Staphylococcus aureus* (*S. aureus* A-100, produces enterotoxin A; *S. aureus* ATCC 14458, produces enterotoxin B; and *S. aureus* 993, produces enterotoxin D – all strains were recovered on Baird-Parker Agar containing egg yolk tellurite and Yeast Extract). For validation testing, the *S. aureus* aqueous suspension was spread onto on agar surfaces, representative of porous materials with hard-to-reach places, such as those found in real-world decontamination applications, and challenged with ClO_2_ solution from D-FENS.

#### 2.2.3. D-FEND ALL and CoD

D-FEND ALL and CoD are chemical systems that eliminate the need for an effector in generating ClO_2_ with two-components and one-step mixing, to provide more convenience in treating water, decontaminating textiles, or for in-package anti-microbial treatments of sterile instruments or fresh fruits and vegetables. The D-FEND ALL and CoD systems use separate oxidation–reduction chemical systems that eliminate the need for the added chemical effector. D-FEND ALL (Doona et al., 2014) was validated experimentally in the laboratory to decontaminate live cultures of *B. anthracis* Delta Sterne (recovered on Nutrient Agar and incubated for 16–20 h at *T* = 35°C) and *Bacillus amyloliquefaciens* spores (recovered on ST-1 Nutrient Agar incubated for 18 h at *T* = 30°C) on military textiles, and compared with commercial household bleach (5–6% aqueous hypochlorite, OCl^-^) and high pressure processing (HPP) for textiles immersed in water using a PT-1 high pressure unit (Avure Technologies, Inc., Kent, WA, USA) with conditions of pressure *P* = 550 MPa, temperature *T* = 65°C, and time *t* = 100 min. CoD was validated for decontamination in rigid plastic packaging using the *G. stearothermophilus* and *B. atrophaeus* spore bio-indicators mentioned above.

#### 2.2.4. FDKs

**FIGURE 4:**
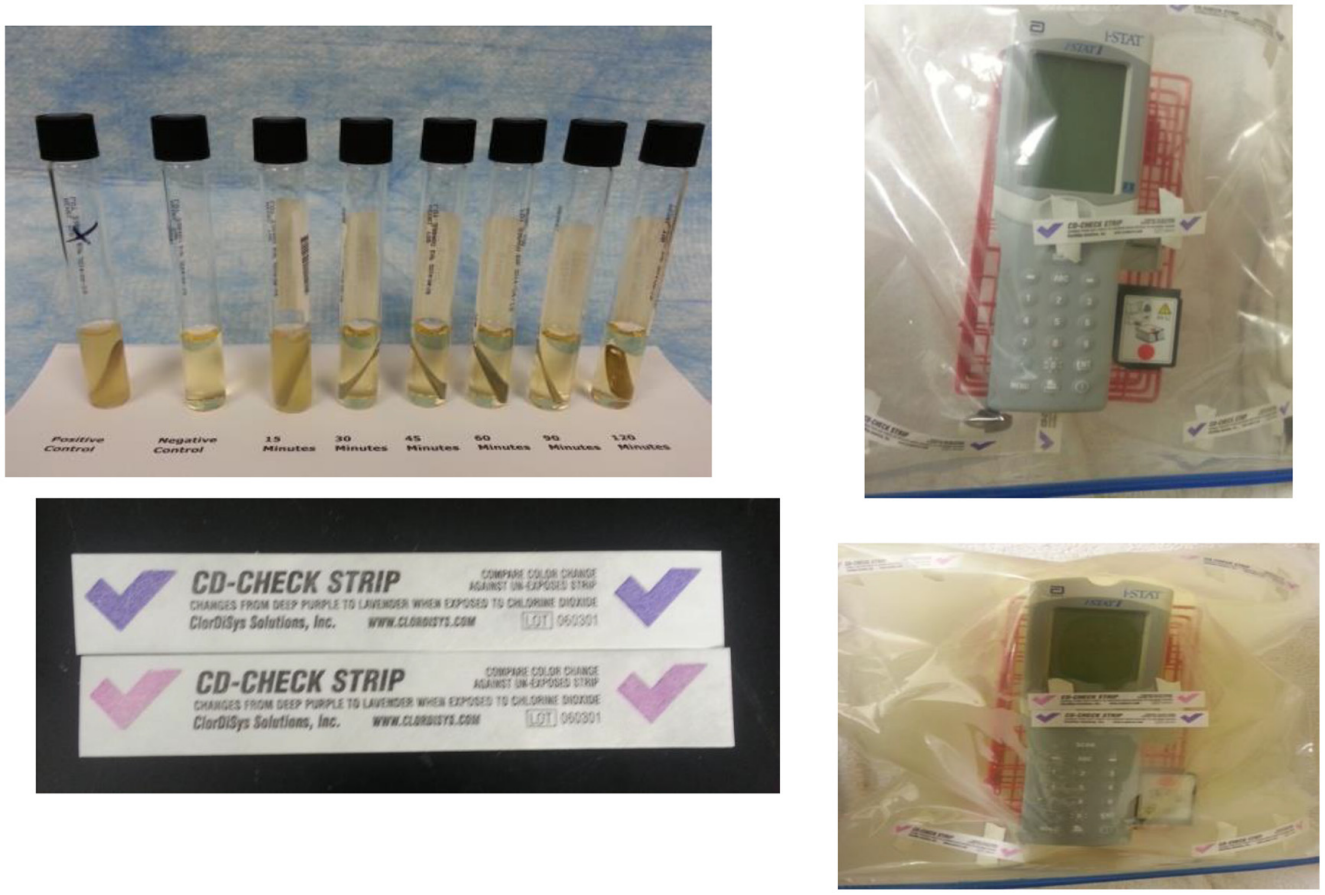
**Laboratory testing showed the FDKs were non-destructive to household electronics devices after 15 cycles (top right) and non-destructive to an iStat device after five decontamination cycles (bottom right).** The FDK used a CHEM-CD configuration (see Section 3) and generated copious ClO_2_ gas **(bottom left)** and *T* = 80–120°C to kill spore indicators in 30 min **(top left)**.

Field Decontamination Kits (FDKs) are based on adapting commercial versions of the NSRDEC decontamination technologies for use by global public health organizations (MSF, WHO, NIH, etc.) to sterilize Ebola-contaminated medical equipment at remote clinical sites in West Africa. First, the NCC is sold commercially as CHEM-CD (ClorDiSys Solutions, Inc., Lebanon, NJ, USA) as a result of Technology Transfer licensing agreements with private industry. CHEM-CD controllably produces gaseous ClO_2_ to decontaminate HEPA housings, biosafety cabinets (BSCs) and hoods, and bio-aerosol chambers. CHEM-CD consists of oxidant (Part A), reductant (Part B), and neutralizer (Part C) in separate foil pouches and wrapped in plastic bag to extend shelf-life to ∼30 months. A video demonstration of CHEM-CD in action is available at http://www.youtube.com/watch?v=EAh_Vz3TNTo. The benefits of using this product relate to the approval of ClO_2_ as a sterilant by the National Sanitation Foundation (NSF/ANSI 49 Annex G 2009) for advantages in safety, speed, and environmental-friendliness compared to conventional formaldehyde. The CHEM-CD product is the method for producing ClO_2_ in the deployed FDKs (**Figure 4**). A more detailed description of the materials and operation of the FDKs are discussed in detail in Section 3. Validation of the PCS using the CHEM-CD formulation from the FDK to sterilize *G. stearothermophilus* and *B. atrophaeus* bio-indicators, as reported previously (Doona et al., 2014), are presentedin Section 3.1 below.

### 2.3. Atomic Force Microscopy (AFM)

Detailed experimental procedures for AFM imaging of spores were described previously (Plomp et al., 2005a,b, 2014) and are summarized here. Droplets (∼2.0 μl) of spore suspensions were deposited on plastic cover slips and incubated for 10 min at room temperature, then the sample substrate was carefully rinsed with double-distilled water and allowed to dry. Images were collected using a Nanoscope IV Atomic Force Microscope (Bruker Corporation, Santa Barbara, CA, USA) operated in tapping mode. For rapid, low-resolution analysis of spore samples, fast scanning AFM probes (DMASP Micro-Actuated, Bruker Corporation, Santa Barbara, CA, USA) with resonance frequencies of ∼210 kHz were utilized. For high-resolution imaging, SuperSharpSilicon (SSS) AFM probes (NanoWorld Inc., Neuchâtel, Switzerland) with tip radii <2 nm and resonance frequencies of ∼300 kHz were used. Nanoscope software 5.30r3sr3 was used for data acquisition and subsequent processing of AFM images. In order to assess low-resolution and high-resolution spore features, raw AFM images typically needed to be modified. In particular, the *contrast enhancement* command, which runs a statistical differencing filter on the acquired image, was typically utilized to bring all of the features of an image to the same height and to equalize the contrast among them. This allows all features of an image to be seen simultaneously, and thus a single spore or a group of spores can be imaged at relatively low resolution while spore coat attributes can be visualized at high-resolution. Heights of spore surface features (i.e., folds, coat layers, etc.) were measured from *height* images using the *section* command. Tapping amplitude, phase, and height images were collected simultaneously. Height images allow quantitative height determinations, providing precise measurements of spore surface topography. Amplitude and phase images do not provide height information, but provide similar morphological and structural information as height images, often displaying a greater amount of structural detail and contrast compared to height images and making them a preferred choice for presentation purposes. Prior to AFM characterization, spore preparations were examined for refractility by phase-contrast light microscopy (Nikon Eclipse 50i) to determine the fraction of ungerminated (phase bright) and germinated (phase dark) spores.

### 2.4. Bacterial Spore Bio-indicators (BI’s)

#### 2.4.1. Spore Strips

Spore strips (Raven Biological Labs, Omaha, NE, USA) are inch-long pieces of cellulose paper inoculated with a known concentration of bacterial spores, and packaged in a barrier material that permits the diffusion of sterilant gas or humidified air, but excludes contaminants such as vegetative bacterial cells or other spores. Spore strips act as bio-indicators (BI’s) for various sterilization/decontamination processes (ethylene oxide, abbreviated EtO, ClO_2_, autoclaving, irradiation, etc.). Three *Bacillus* spore species were assayed for susceptibility to ClO_2_: *B. atrophaeus* (ATCC 9372, formerly *B. subtilis* var. niger and *B. globigii*), *B. thuringiensis* (ATCC 29730), and *G. stearothermophilus* (ATCC 7953, formerly *Bacillus stearothermophilus*). Spore strip populations for all species were determined by the manufacturer, as were D and Z (the slope of a thermal resistance curve) values for two of the three species. D values for ethylene oxide (D_EtO_) and dry heat (D_160_) were determined for *B. atrophaeus*, while D values for saturated steam (D_121_ and D_132.2_) were determined for *G. stearothermophilus*. No such values were determined for *B. thuringiensis*. Species lot numbers, populations, and relevant D and Z values are listed in **Table 2**.

**Table 2:** Spore strip bio-indicator characteristics.

Species	ATCC#	Lot#	Population	D_160_ value	D_EtO_ value	*Z*-value
*Bacillus atrophaeus*	9372	1162052	2.0 × 10^6^	2.6	3.0	44.5
*B. atrophaeus*	9372	1161841	1.3 × 10^6^	2.5	3.3	23.5
*B. atrophaeus*	9372	1161911	1.3 × 10^6^	2.5	3.8	46.0
**Batch 204GB:**						
*B. atrophaeus*	9372	1142042	1.2 × 10^4^	2.8	3.1	39.0
*B. atrophaeus*	9372	1152041	1.2 × 10^5^	2.8	3.1	39.0
*B. atrophaeus*	9372	1162043	1.2 × 10^6^	2.8	3.1	39.0
*B. atrophaeus*	9372	1172041	1.2 × 10^7^	2.8	3.1	39.0
**Batch 214GB:**						
*B. atrophaeus*	9372	1142141	3.5 × 10^4^	2.8	5.0	33.3
*B. atrophaeus*	9372	1152141	2.0 × 10^5^	2.8	5.0	33.3
*B. atrophaeus*	9372	1162141	1.5 × 10^6^	2.8	5.0	33.3
*B. atrophaeus*	9372	1172141	1.5 × 10^7^	2.8	5.0	33.3
*B. atrophaeus*	9372	1182141	1.5 × 10^8^	2.8	5.0	33.3
*B. thuringiensis*	29730	616022	1.2 × 10^6^	n/a	n/a	n/a
				**D_121_ value**	**D_132.2_ value**	**Z-value**
*Geobacillus stearothermophilus*	7953	3166031	1.0 × 10^6^	2.0	0.07	7.5


#### 2.4.2. BI Packaging Material

The BI packaging material contributes a crucial factor in the efficacy of the ClO_2_ sterilizing process. In this report, we investigate BI’s packaged either in 1059B medical grade Tyvek (single-sided with a plastic backing; Raven Industries, Sioux Falls, SD, USA) or nothing, as our preliminary data suggested that 1059B was relatively non-reactive and non-attenuating for ClO_2_. To comply with current industry standards, we also show that ClO_2_ is an efficacious sterilant of *B. atrophaeus* BI’s packaged in medical grade glassine (Raven Industries, Sioux Falls, SD, USA).

#### 2.4.3. ClO_2_ Generation and Treatment

Chlorine dioxide was generated by the oxidation of technical grade sodium chlorite (NaClO_2_) by sodium persulfate (Na_2_S_2_O_8_; Sigma-Aldrich Co., St. Louis, MO, USA) in aqueous solution:

$$2NaClO_{2}+{Na}_{2}S_{2}O_{8}\to 2\,\;ClO_{2}+2{Na}_{2}SO_{4}$$

Pure gaseous ClO_2_, free of volatile by-products such as Cl_2_, was purged from the reaction flask and diluted with ratios of filtered, dehumidified and humidified air to attain the target ClO_2(g)_ concentration and RH conditions. RH was controlled with a series of flow meters (Cole Parmer, Vernon Hills, IL, USA) passing filtered and dehumidified air through a 500 mL gas wash bottle half filled with deionized water. The humidified diluted gas was directed into a 5 L glass test chamber (Thermo Fisher Scientific, Waltham, MA, USA), and ClO_2_ concentration ([ClO_2_]) in parts per million (ppm) was determined by iodometric titration of a 50 or 100 mL volume/sample of gas taken/removed/sampled from the exit port of the reaction chamber in a gas-tight Hamilton sample-lock syringe (Fisher). Experiments were carried out at ambient temperature and atmospheric pressure (measured and monitored with a Traceable^®^ Digital Hygrometer/Thermometer (VWR International, Radnor, PA, USA), with RH ranging from 30 to 90%, and [ClO_2_] ranging from approximately 50 to 2000 ppm. Experimentally measured values for temperature, RH and [ClO_2_] are listed in **Table 3**.

**Table 3:** Experimental ClO_2_ target and actual concentrations.

ClO_2_ concentration (ppm)			ClO_2_ dose			%RH		
Target	Measurement	SD	Target	Measurement	SD	Target	Measurement	SD
50	50	4	400	414	20	30	30.32	0.006
67	68	4	1000	1037	32	40	38.75	0.011
100	110	17	2000	2020	40	50	49.51	0.015
125	131	6	4000	4046	88	60	59.41	0.013
167	177	21				70	69.36	0.011
200	218	14				80	79.42	0.015
250	256	23				90	89.67	0.01
400	405	31						
500	498	40						
800	784	53						
1000	1027	115						
2000	2027	303						


*Room temperature = 21.5°C, SD = 0.770*.

To ensure exposure and contact of BIs with ClO_2_, no more than 20 spore strips were placed in the test chamber at one time, and, thus, multiple runs at each reported dose were performed in order to (i) assure repeatability of our results, and (ii) gather enough data to achieve statistically significant results (**Table 4**). One negative control strip (no inoculum and packaged appropriately) was included in the chamber for each set of 10 test strips, and a positive control strip remained outside of the test chamber and away from other potential sterilizing agents for the duration of each experiment.

**Table 4:** Tyvek spore strip data summary.

Species	Runs		*N*	Dose	%RH	D_EtO_
	Tyvek	No pkg		(ppm ClO_2_× t)		
*Bacillus atrophaeus*	152	144	1.2 × 10^4^–1.0 × 10^6^	110–1991	79	3.1
*B. atrophaeus*	490	350	3.5 × 10^4^–1.5 × 10^8^	110–1991	79	5.0
*B. atrophaeus*	500	500	1.3 × 10^6^	438–4106	30–90	3.3
*B. atrophaeus*	498	495	1.3 × 10^6^	438–4106	30–90	3.8
*B. thuringiensis*	98	98	1.2 × 10^6^	438–4106	79	–
*G. stearothermophilus*	80	80	1.0 × 10^6^	438–4106	79	–


#### 2.4.4. Microbiological Assays of Sterility

After exposure to the appropriate ClO_2_ dose, the strips were placed, using aseptic technique, into tubes of Tryptic Soy Broth containing a Bromocresol Purple pH indicator (Raven Industries, Sioux Falls, SD, USA) and incubated at 37°C (*B. atrophaeus* and *B. thuringiensis*) or 60°C (*G. stearothermophilus*) for 7 days. We monitored the tubes on a daily basis for both turbidity (indicative of bacterial growth) and color change of the pH indicator from purple to yellow (indicative of metabolism). Criteria for a strip being considered “killed” were findings of both *no* turbidity and of *no* color change of the pH indicator, coupled with growth and metabolism for the positive control associated with the sample test set and no growth for the negative control.

#### 2.4.5. Statistical Analysis and Modeling

We fitted a binomial generalized linear model with a complementary log-log link function to the proportion of strips still having live spores after treatment, allowing the dispersion parameter to be greater than one to account for over-dispersion. We adjusted for covariates such as (logarithm of) the number of spores on a strip, the type of packaging used to store the spore strips, RH, and D_EtO_ values. The model in its most general form is expressed as Eq. (1)

(1) $$\log\left(-\log\left(1-Ø\right)\right)=b_{0}+b_{1}*Dose+b_{2}*\log N+b_{3}*Pack+b_{4}*RH+b_{5}*D_{EtO}\,\;\left(1\right)$$

in which Ø is the probability of a strip still having living spores after treatment, *p_N_* is the probability of a spore remaining alive after treatment (depends on *N*), *Dose* is the dose of ClO_2_ as calculated by ClO_2_ ppm multiplied by exposure time (in units of hours), *N* is the number of spores on a strip, *Pack* equals 1, if spore strips came in Tyvek packaging, and *Pack* equals 0, if there were no packaging, *RH* is the relative humidity (RH; as a proportion), *D_EtO_* is the time (in units of minutes) to reduce the number of living spores to 10% of the original value by ethylene oxide (**Table 5**), and *b*’s are regression coefficients. The relationship between the strip survival probability Ø and the spore survival probability *p_N_* is expressed in Eq. (2)

(2) $$1-Ø={\left(1-p_{N}\right)}^{N}\,\;\left(2\right)$$

and Eq. (1) can be re-written as Eq. (3)

(3) $$\log(-\log\left(1-p_{N}\right))=b_{0}+b_{1}*Dose+(b_{2}-1)*\log N+b_{3}*Pack+b_{4}*RH+b_{5}*D_{EtO}\,\;\left(3\right)$$

**FIGURE 5:**
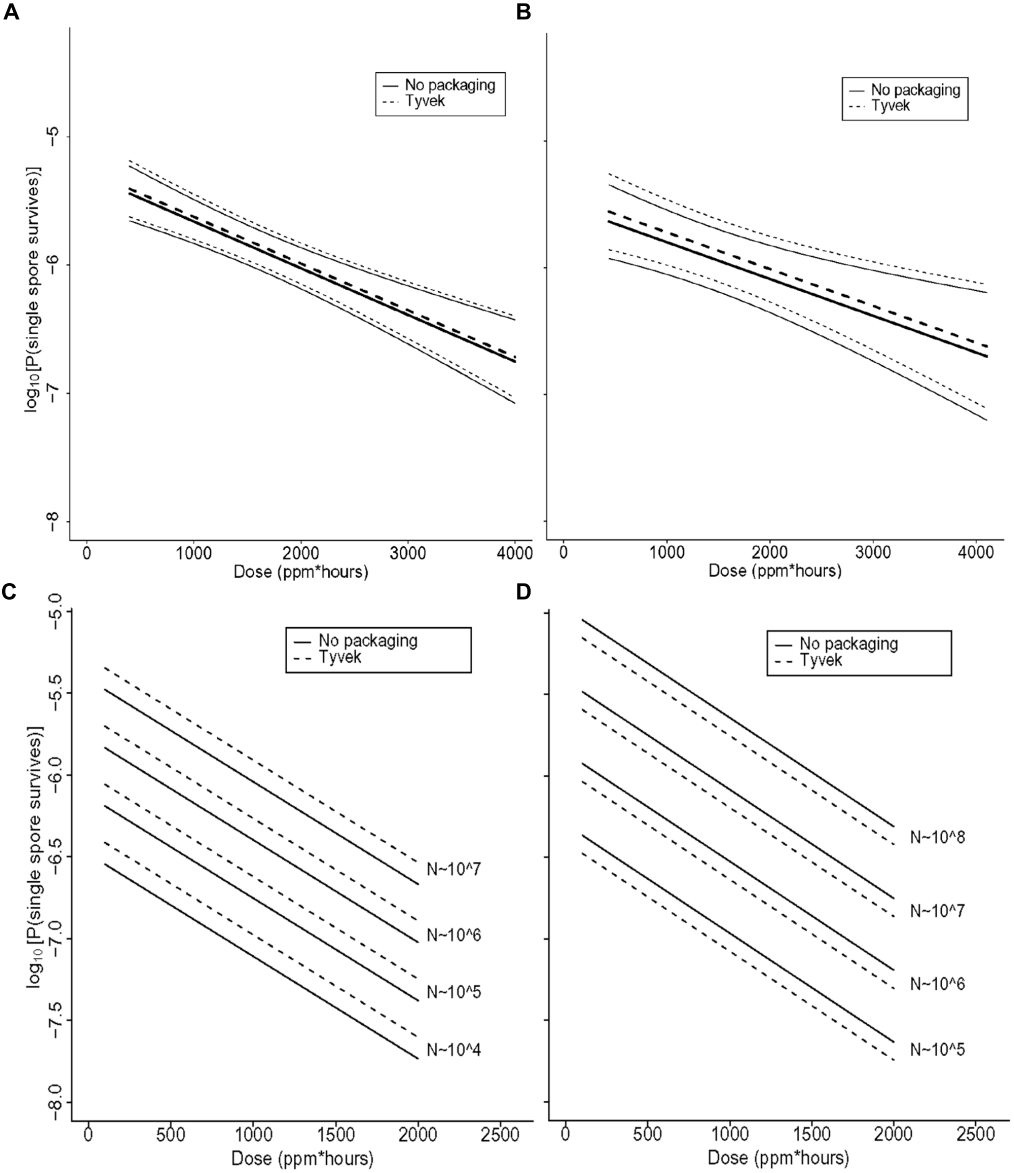
**(A)** Statistical modeling showing the probability, with 95% confidence, of a single *B. thuringiensis* spore surviving a ClO_2_ dose range of 438–4106 ppm-h at 79% RH. The spore population of each strip is fixed at 1.2 × 10^6^, and *n* = 98 each for strips contained in Tyvek or with no packaging. In the figure, the lighter set of dashed and solid lines represent the confidence interval for spore strips packaged in Tyvek or no packaging, respectively, while the middle bold lines represent the predicted probabilities. In this model, there is strong evidence of an effect of dose (*p* = 0.002) and no evidence of an effect of packaging (*p* = 0.76). **(B)** Statistical modeling showing the probability, with 95% confidence, of a single *G. stearothermophilus* spore surviving a ClO_2_ dose range of 438–4106 ppm-h at 79% RH. The spore population of each strip is fixed at 1.0 × 10^6^, and *n* = 80 each for strips contained in Tyvek or no packaging. Lighter dashed and solid lines represent the confidence interval for spore strips packaged in Tyvek or no packaging, respectively, while the middle bold lines represent the predicted probabilities. There is evidence of an effect of dose (*p* = 0.02) and no evidence of an effect of packaging (*p* = 0.68). **(C)** Predicted probabilities of a single spore surviving for varying numbers of *B. atrophaeus* spores (1.2 × 10^4^ to 1.2 × 10^7^), with a D_EtO_ value of 3.1, in Tyvek (*n* = 152) and no packaging (*n* = 144) at 79% RH and ClO_2_ dose ranging from 110 to 1991 ppm-h. There is strong evidence that increased dose decreases the probability of any strips having live spores (*p* < 0.01) and that as the number of spores on the strip increases, so does the probability of survival (*p* < 0.01). There is no evidence of a significant effect of packaging on survival of spores (*p* = 0.21), though there is some difference with a slightly higher rate of survival for spores packaged in Tyvek. **(D)** Predicted probabilities of a single spore surviving for varying numbers of *B. atrophaeus* spores (3.5 × 10^4^ to 1.5 × 10^8^), with a D_EtO_ value of 5.0, in Tyvek (*n* = 490) and no packaging (*n* = 350) at 79% RH and ClO_2_ dose ranging from 110 to 1991 ppm-h. These results indicate that the probability of a strip having live spores after treatment increases with decreasing dose (*p* < 0.01) and with increasing numbers of spores (*p* < 0.01). There is no evidence of an effect of packaging (*p* = 0.21), although there is a slightly lower rate of survival for spores in Tyvek packagi.

**FIGURE 6:**
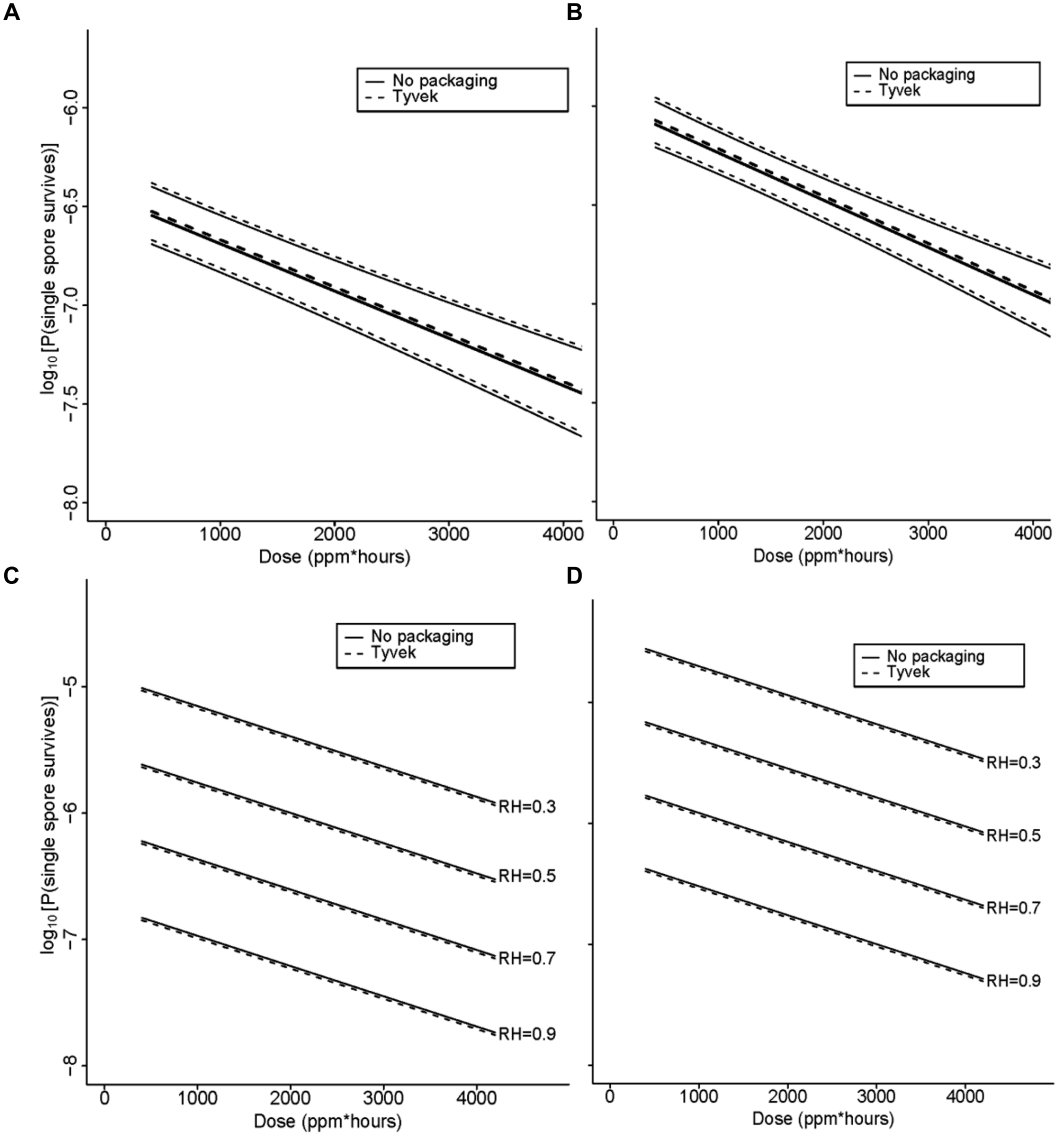
**(A)** Statistical modeling showing the probability, with 95% confidence, of a single *B. atrophaeus* spore, with a D_EtO_ value of 3.1 and fixed strip inoculum of 10^6^, in Tyvek (*n* = 38) and no packaging (*n* = 36), surviving a ClO_2_ dose range of 110–199 ppm-h at 79% RH. In the figures **(A–D)**, the lighter set of dashed and solid lines represent the confidence interval for spore strips packaged in Tyvek or no packaging, respectively, while the middle bold lines represent the predicted probabilities. **(B)** Statistical modeling showing the probability, with 95% confidence, of a single *B. atrophaeus* spore, with a D_EtO_ value of 5.0 and fixed strip inoculum of 10^6^, in Tyvek (*n* = 98) and no packaging (*n* = 70), surviving a ClO_2_ dose range of 110–1991 ppm-h at 79% RH. **(C)** Predicted probabilities for a single *B. atrophaeus* spore, with a D_EtO_ value of 3.3 and fixed strip inoculum of 1.3 × 10^6^, in Tyvek (*n* = 500, dashed line) or no packaging (*n* = 500, solid line). **(D)** A D_EtO_ value of 3.8 and fixed strip inoculum of 1.3 × 10^6^, in Tyvek (*n* = 498, dashed line) or no packaging (*n* = 498, solid line), surviving a range of ClO_2_ doses with RH varying from 30 to 90%. The results of this fitted model, which has an offset term of log(N; fixed in this example) shows again that dose has a similarly strong effect as previously in that increasing it decreases the probability of spores surviving on a strip (*p* < 0.01). There is no evidence of an effect of packaging (*p* = 0.73), and there is strong evidence of an increase in survival probability with increasing D_EtO_ values (*p* < 0.01) and with decreasing RH (*p* < 0.01). The D_EtO_ variable was treated as a categorical variable to be consistent with earlier models and so the regression coefficient compares D_EtO_ = 3.3 to D_EtO_ = 3.8.

**FIGURE 7:**
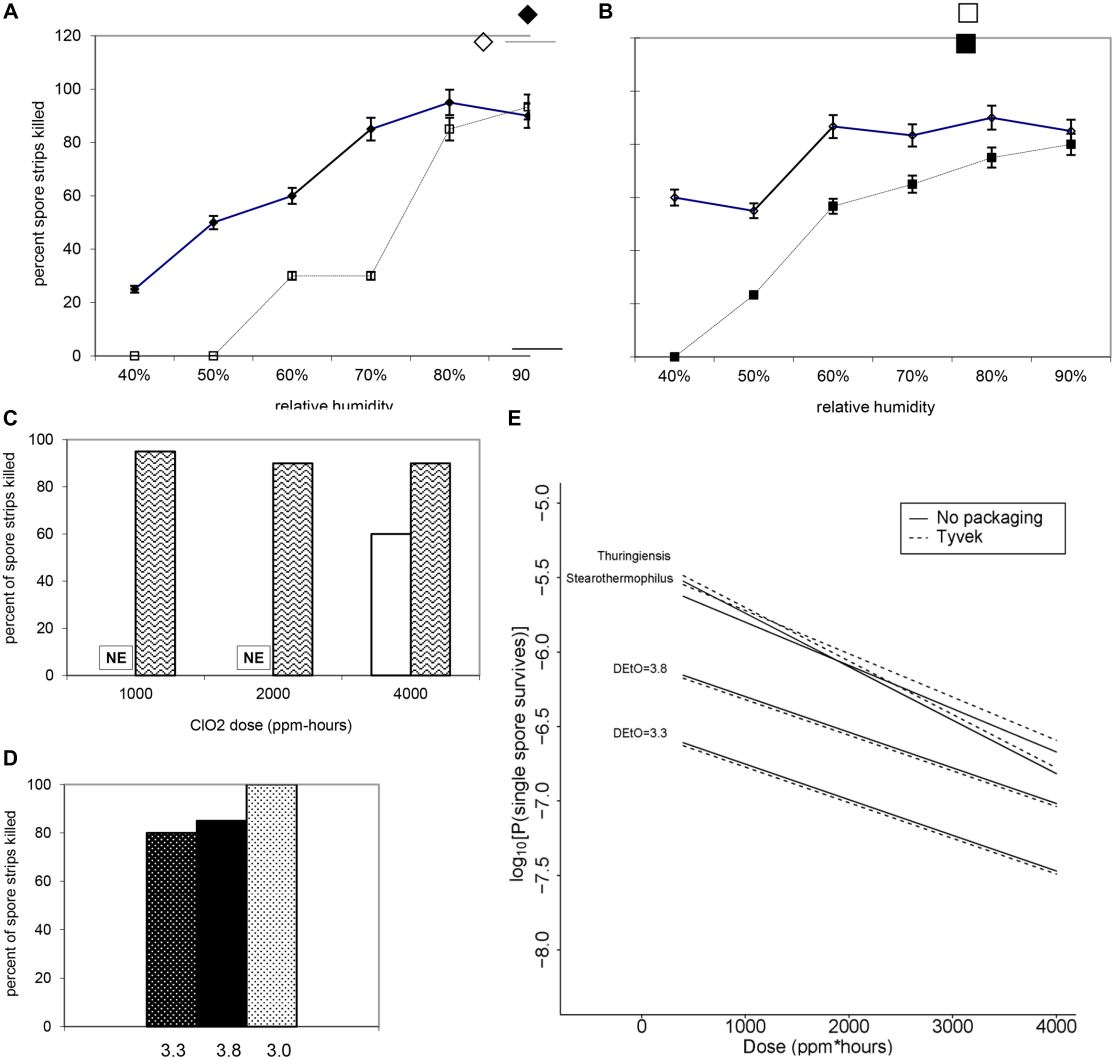
**Increasing RH at a fixed dose of **(A)** 500 ppm ClO_2_ for 4 h (∼2000 ppm-h) and **(B)** 1000 ppm ClO_2_ for 4 h (∼4000 ppm-h) results in a higher percentage of log 6 *B. atrophaeus* spore strips being killed.** **(A)** *B. atrophaeus* D_EtO_ = 3.3 (*n* = 120), = *B. atrophaeus* D_EtO_ = 3.8 (*n* = 160); **(B)** *B. atrophaeus* D_EtO_ = 3.3 (*n* = 140), and *B. atrophaeus* D_EtO_ = 3.8 (*n* = 140). Data shown is for spore strips packaged in Tyvek. For the D_EtO_ = 3.3 strips exposed to ∼2000 ppm-h ClO_2_, ≥80% kill is achieved at 70 %RH and above, but when we increase the dose to ∼4000 ppm-h the same level of kill is achieved at %RH of 60. The relationship between low D_EtO_ value and increased susceptibility to ClO_2_ is evident, though, in this example, we see that at 90 %RH all four sample sets are behaving roughly the same. **(C)** *B. atrophaeus* D_EtO_ = 3.3, log 6 spore strips packaged in img medical-grade glassine, and img Tyvek are not equally susceptible to a set ClO_2_ dose at 79 %RH (NE = no effect). Spore strips were exposed to 250 ppm ClO_2_ for 4 h (dose ≈1000 ppm-h), where *n* = 20 each for glassine and Tyvek; 2000 ppm-h, where *n* = 40 (glassine) and *n* = 20 (Tyvek); and 4000 ppm-h, where *n* = 40 (glassine) and *n* = 20 (Tyvek) at constant temperature and %RH. **(D)** We did not achieve 100% kill for glassine-packaged log 6 spore strips until we subjected a lot of *B. atrophaeus* with D_EtO_ value of 3.0 (*n* = 20) to 2000 ppm for 10 h (∼20,000 ppm-h) at 79 %RH. This high dose killed 80 and 85% of *B. atrophaeus* D_EtO_ = 3.3 and 3.8 (*n* = 20 each) spore strips, respectively. **(E)** For all three species and *B. atrophaeus* with two different D_EtO_ values (3.3, with *n* = 80 for each Tyvek and no packaging; and 3.8, with *n* = 189 for each Tyvek and no packaging), probabilities were predicted for a spore surviving after treatment, with RH = 0.79 and *N* = 10^6^. Note that for *B. thuringiensis* (*n* = 98 each for Tyvek and no packaging), measurements were only made at *N* = 1.2 × 10^6^. For both sets of *B. atrophaeus*, measurements were made at *N* = 1.3 × 10^6^, so in these cases this plot involves extrapolation in *N*. *B. thuringiensis*, the closest phylogenetic relative to *Bacillus anthracis* used in this study, is more likely to survive at any given ClO_2_ dose than either of the *B. atrophaeus* strains, though it’s resistance is similar to the strain of *G. stearothermophilus* (*n* = 80 each for Tyvek and no packaging) assayed.

The range of values of each of the covariates in the various experiments is described in (**Table 4**). Although the independent variable is the strip survival probability Ø, **Figures 5–7** are reported in terms of our primary measure of interest, which is the probability *p_N_* that any single spore survives.

## 3. Results – Field Decontamination Kits in the Ebola crisis

Emerging and re-emerging viral infectious diseases have been identified as a major threat to human health, and the current outbreak of EVD has impacted a large part of Western Africa. Conventional procedures for the decontamination of equipment during a filovirus outbreak rely on the use of chemical chlorine (bleach, aqueous hypochlorite, OCl^-^) for decontamination when exiting an isolation/treatment center. Electrical equipment that enters an isolation/treatment center and diagnostic facilities can only be surface decontaminated by aqueous rinses with 0.5–5.0% chlorine bleach solution (household bleach is about 6% OCl^-^). This procedure is likely *not* adequate for complete decontamination of the devices, particularly for accessing inside the devices.

Devices such as personal cell phones, computers, and most importantly, expensive medical point-of-care (POC) electronic devices used for clinical assessment of patients in isolation/treatment ward are not sufficiently decontaminated by surface rinses. Some medical diagnostic devices have closed systems internal to the device that may have infrequent but probable contamination that cannot be adequately decontaminated by surface rinses with a chemical decontaminating agent. Often these devices contain valuable internal components that may be recycled after being surface decontaminated. This is extremely problematic for items used in a high hazard environment. For low resource, remote sites, the deployment of traditional decontamination tools is nearly impossible. Often there is limited space for equipment, so another desirable aspect of the FDK method is its compact size and simple disposal routine post-decontamination cycle.

**FIGURE 8:**
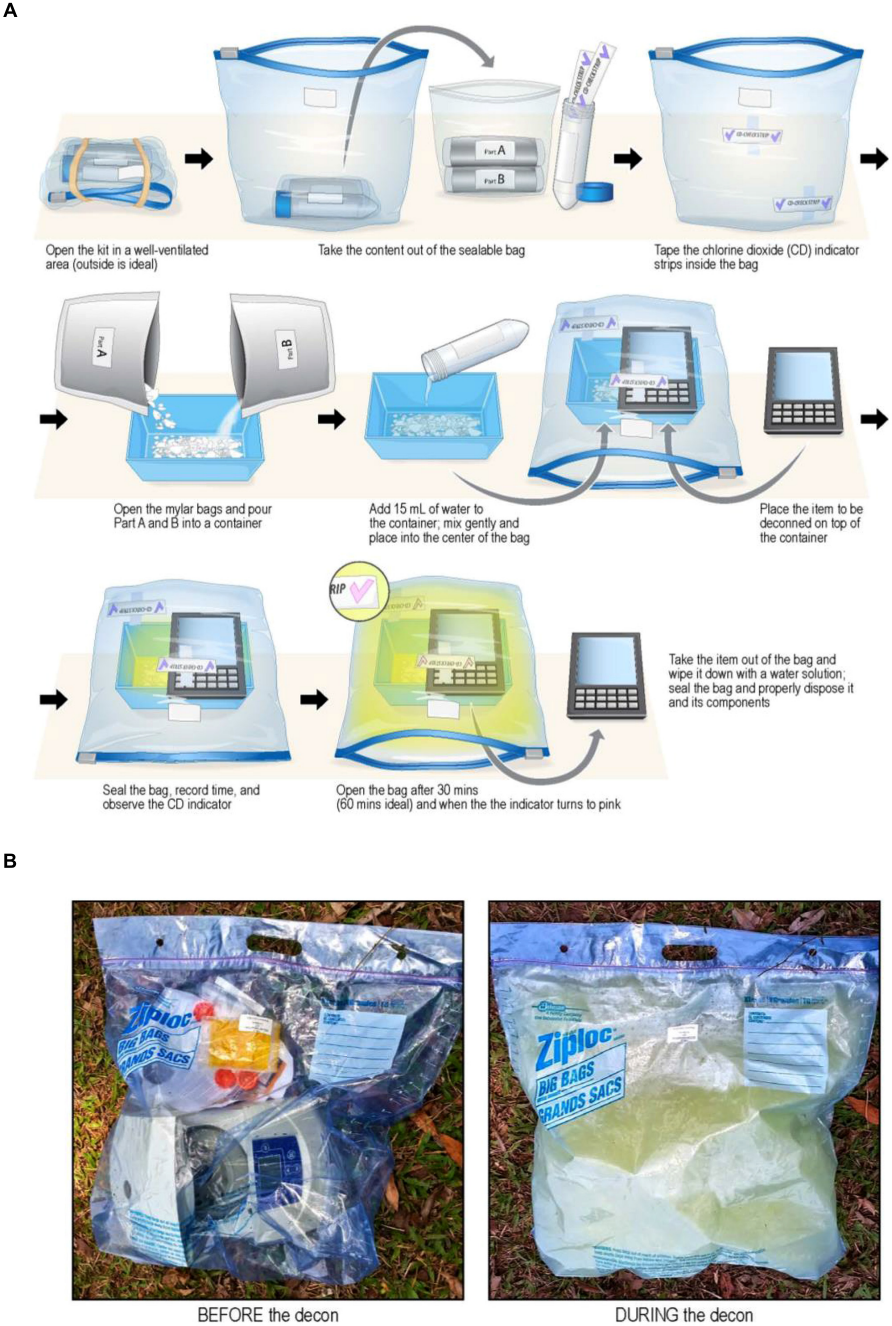
**(A)** Diagrammatic representation of steps for using FDKs. Top Row. (i) Open FDK in well-ventilated area (outdoors), (ii) remove contents form sealable bag, and (iii) tape ClO_2_ indictor strips to inside of bag. Middle Row: (iv) Empty foil pouches of chemical reagents (Part A and Part B) into plastic container, (v) add 15 mL of water with mixing, and (vi) place plastic container inside bag, then place contaminated medical device on top of container. Bottom Row: (vii) Seal bag, run for 30–60 min, and observe purple → pink color change of ClO_2_ indicator strip, then (viii) with gloves on, open bag, remove device from bag, and re-seal bag for proper disposal. Caution: when opening bag, gas has a strong odor – avoid inhaling gas. With gloves on, use water to wipe residue from device/equipment (Illustration provided courtesy of Mr. Jiro Wada of NIH). **(B)** Images of an actual decontamination procedure (before and during) of a microcentrifuge with an FDK on the ground in Liberia (Photos courtesy of Dr. Lisa Hensley and Mr. James Pettitt of NIH/NIAID).

Given the wide use of and acceptability of chlorine bleach solutions in laboratories and in isolation/treatment centers, and the historical use of chlorine dioxide in the decontamination of equipment in the U.S., we decided to adapt a chlorine dioxide method to provide equipment decontamination for deployment in the field in areas of active outbreak. This safe and adaptable method uses a commercially available chemical combination kit called CHEM-CD (ClorDiSys Solutions, Inc., Lebanon, NJ, USA) in which the components are packaged separately and isolated from water to prevent chemical reaction from taking place (**Figure 8A**). Once mixed together with water, the chemical reduction of sodium chlorite initiates within minutes, and a well-controlled, exothermic reaction takes place that releases chlorine dioxide gas from solution. This reaction is carefully designed to occur inside a closable plastic bag, such that the ClO_2_ released decontaminates thoroughly the entire interior volume of the bag and all items contained therein, including permeating the interior regions of the electronic medical and other devices inside the decontamination bag. Humidity (≥70% RH) and mild heat and mild pressure accumulate inside the bag from the chemical reaction occurring inside a closed container, but the heat and pressure gently subside over the 30–60 min decontamination period. Human exposure to ClO_2_ above permissible concentrations and durations is known to cause irritation of the eyes, skin, nose, throat and lungs, and thus this method is typically used in outdoor environments with exposure to the sun.

Adapting this method for field decontamination was ideal, because it is devoid of electrical power sources, uses small quantities of water from available sources, and the low cost of the FDKs, and with the required supplies simple and easy to obtain, store, and handle for ClO_2_ generation. Specifically, this method was used to develop FDKs (**Table 1**; **Figure 8B**) comprising a self-sealable bag (2 or 10 gallon capacity), dry chemical components (Part A and Part B from the commercially available CHEM-CD kit) that react in water to produce chlorine dioxide. The FDK also includes a device to measure water (50 mL tube) and ClO_2_ indicators of sterility (CD Check Strip, ClorDiSys Solutions, Inc., Lebanon, NJ, USA). In addition, laboratory testing with spore BIs (*G. stearothermophilus* for steam sterilization from BT Sure, Thermo Scientific, Marietta, OH, USA, and *B. atrophaeus* for ethylene oxide sterilization from EZ Test, SGM Biotech, Inc., Bozeman, MT, USA as discussed above) showed complete inactivation of spores (sterility) was achieved within 30 min of exposure.

**Figure 8A** illustrates the procedural steps carried out in utilizing the FDKs. Briefly, the ClO_2_ indicators are taped to the inner bag to be observed during the decontamination process and the chemical reagents Part A and Part B are placed in a container with water. The item to be decontaminated is placed in the bag near or over the mixture and the bag is subsequently sealed. Within a few minutes the reaction produces condensation (humidity) and yellow ClO_2_ gas that inflates the bag due to a mild build-up of heat and pressure, although the bag itself does *not* become turgid. After a minimum of 30 min, the color change of the indicator shows adequate concentration and exposure to ClO_2_ gas to achieve sterility, and the bag can be opened to release the trapped gas. The equipment can then be removed and surface-cleaned, since the water, ClO_2_, and inert salts from the spent reaction can be present on the surface. The remaining solution and materiel can be bagged and disposed of according to local policies.

More than 150 FDKs have been deployed for use by global public health organizations during the Ebola outbreaks in Western Africa, which includes Guinea, Sierra Leone, and Liberia, to protect patients and health care personnel. The FDKs have been used to decontaminate laboratory equipment, POC medical devices and personal cell phones. Overall, the performance has been acceptable and only on occasion have the bags leaked gas during the sterilization cycle, thereby requiring longer exposure times to effectuate the color change of the ClO_2_ indicator strip and signify exposure sufficient to ensure sterility. For the purpose of field deployment, these kits are safe, compact and easy to ship to remote sites that have limited infrastructure and resources available.

### 3.1. Results – Laboratory Testing NIH/NIAID Field Decontamination Kits

Instructions for the FDKs involve putting Part A and Part B together into a reaction vessel, then adding water. Independent laboratory testing showed that increasing the volume of water slightly and adding the water to the reaction vessel first, followed by adding Part A (oxidant) with stirring, then Part B (reductant) with stirring helped ensure the reaction runs controllably and smoothly, while evolving the maximum amount of ClO_2_ gas. Small FDKs consist of a 2.5 gallon sealable Mylar bag, 15 g of Part A, 4 g of Part B, and 30 mL of water mixed inside a 100 mL beaker as the reaction vessel. Large FDKs were scaled proportionately to consist of a 10-gallon bag, 60 g of Part A, 16 g of Part B, and 120 mL of water in a 600 mL beaker as the reactor vessel. In both configurations, the chemical reaction initiates slowly, then releases copious gaseous ClO_2_ at ∼2 min and 15 s after all of the reagents were combined. These reaction compositions with various permutations were run inside the different FDKs (2.5- and 10-gallon bags) and also inside the PCS, using spectrophotometry to monitor [ClO_2_] at λ = 360 nm (the absorbance maximum of ClO_2_) and a combination probe that measures %RH and temperature simultaneously (6621 – Commercial HVAC Temperature/RH Transmitter, Testo, Inc., Sparta, NJ, USA). In addition, the reaction was monitored with ClO_2_ color-change indicator strips (CD-CHEK, ClorDiSys Solutions, Inc., Lebanon, NJ, USA) and commercially available spore indicators of *G. stearothermophilus* in Tyvek (ClorDiSys Solutions, Inc., Lebanon, NJ, USA), *G. stearothermophilus* for steam sterilization (BT Sure, Thermo Scientific, Marietta, OH, USA), and *B. atrophaeus* for ethylene oxide sterilization (EZ Test, SGM Biotech, Inc., Bozeman, MT, USA). Representative results are summarized in **Table 5** using the commercial chemical sets and the three different container units (2.5-gal bag, 10-gal bag, and PCS) run for 30 min. In all instances, the CD-CHEK strips turned color (indicative of sufficient ClO_2_ exposure for sterilization) and all of the spore bio-indicators confirmed sterility had been achieved.

**Table 5:** Laboratory tests of FDKs and the PCS.

Test code	Container	Conditions	Observations	Microbiological Results
Test a	2.5-gal bag	- 15 g Part A	Reaction in 2:20	Sterilized_a,b_
		- 4 g Part B	RH >96.4%	
		- 30 mL H_2_O (tap)	*T* = 30°C	
		in a 100 mL beaker	[ClO_2_] >7000 ppm	
Test h	10-gal bag	- 15g Part A	Reaction at 2:10	Sterilized_a,b_
		- 4 g Part B	RH >74%	
		- 30 mL H_2_O (tap)	*T* = 25.4°C		
		in a 100 mL beaker		
Test i	PCS	- 16 g Part A	Reaction in 2:30	Sterilized_a,b_
		- 4 g Part B	RH >96.2%	
		- 30 mL H_2_O (tap)	*T* = 24.4°C	
		in a 100 mL beaker	[ClO_2_] >7000 ppm	
Test n	PCS	- 15g Part A	Reaction at 2:12	Sterilized_a,b_
		- 4 g Part B	RH >93.5%	
		- 30 mL H_2_O (tap)	[ClO_2_] >7000 ppm	
		in a 100 mL beaker	Run time 15 min	

*^a,b^Sterility confirmed with *G. stearothermophilus* and *B. atrophaeus* bio-indicators, respectively*.

### 3.2. Results – Decontamination of Textiles

Some textile technologies have certain material properties with the potential to self-decontaminate by inactivating biological hazards (*B. anthracis* spores) and/or chemical agents on their surfaces, thus making them a form of novel decontamination technology. Other types of textiles require external applications to remove biological and chemical hazards, such as the use of sorbents to remove chemical agents or the use of cold sterilants (bleach, chlorine dioxide, etc.) to safely inactivate biological hazards.

Natick Soldier RD&E Center’s novel decontamination technologies (**Table 1**) have also been applied in the decontamination of textiles using live cultures of *B. anthracis* Delta Sterne as a surrogate for *B. anthracis*. Specifically, NSRDEC’s “D-FEND ALL” technology was used to create 20–200 ppm ClO_2_ solutions that completely inactivated 7.5 logs of *B. anthracis* Sterne inoculated onto two different types of fabrics (a nylon-cotton blend and an experimental weatherproof fabric) within 10 min (minor bleaching of the weatherproof fabric occurred at only the 200 ppm ClO_2_ solution). Other experimental fabrics were inoculated with *B. anthracis* Delta Sterne spores, then subjected to either (i) 30 min rinse in bleach (5–6% aqueous OCl^-^), (ii) gaseous ClO_2_ using NSRDEC’s NCC and PCS technologies (**Table 1**), or (iii) HPP. In all cases, the decontamination treatments achieved a 100% spore kill on the fabric samples (a 6.59-, 5.73-, and 6.13-log kill for bleach, gaseous ClO_2_, and HPP, respectively). For HPP experiments, inoculated fabric samples were treated with conditions of pressure = 550 MPa, temperature = 65°C, and time = 100 min.

### 3.3. Results – AFM Characterization of Morphological and Structural Attributes of *Bacillus* spores

Atomic force microscopy can be used to analyze high-resolution architecture, assembly, structural dynamics, and function of dormant and germinating spores of various wild type and mutant *Bacillus* (Plomp et al., 2005a,b,c, 2007a, 2014; Carroll et al., 2008; Ghosh et al., 2008; Plomp and Malkin, 2009; Malkin and Plomp, 2010; Malkin, 2011; Elhadj et al., in preparation) and *Clostridium* species (Plomp et al., 2007b). Specifically, AFM has been used to directly visualize and analyze spore morphological, dimensional, and high-resolution coat structural attributes, and these results demonstrated that spore morphological and coat structures are phylogenetically (Plomp et al., 2005a,b,c, 2014) and growth medium (Malkin, 2011; Elhadj et al., in preparation) dependent. We have found that strikingly different species-dependent spore coat structures are a consequence of nucleation and crystallization mechanisms that regulate the assembly of the outer spore coat (Plomp et al., 2005a,b,c, 2007b; Malkin and Plomp, 2010). Spore morphological, dimensional, and structural attributes could serve as a baseline for assessing the effects of sterilization and/or decontamination treatments on the morphological and structural integrity and the ultra-structural damage of spores treated by irradiation or ClO_2_.

#### 3.3.1. AFM Characterization of γ-Irradiated *Bacillus* Spores

##### 3.3.1.1. Native air-dried *Bacillus* spore

Size distributions (spore height/width and length) from large populations (several 100s) of air-dried solution- and agar-grown spores were determined for spores of *B. atrophaeus*, *B. thuringiensis*, *B. subtilis* (Plomp et al., 2005a) and *B. anthracis* spores (Elhadj et al., in preparation), with more than 30 spore preparations being utilized for the assessment of *B. anthracis* spore dimensional attributes (Elhadj et al., in preparation). Representative results are compiled in **Table 6**.

**Table 6:** Spore height determinations.

Spore species	Spore height air-dried (solution-grown)	Spore height air-dried (agar-grown)
*Bacillus thuringiensis*^∗^	750–1000 nm	740–1080 nm
	avg ≈ 872 nm	average ≈ 937 nm
	(AD = 5.4%)	(AD = 5.3%)
	(AD = 5.4%)	(AD = 5.3%)
*B. anthracis*^‡^	800–880 nm	750–800 nm
	average ≈ 835 nm	average ≈ 780 nm
	(AD = 5.4%)	(AD = 5.4%)

*^∗^Plomp et al., 2005a; ^‡^Elhadj et al., in preparation*.

**FIGURE 9:**
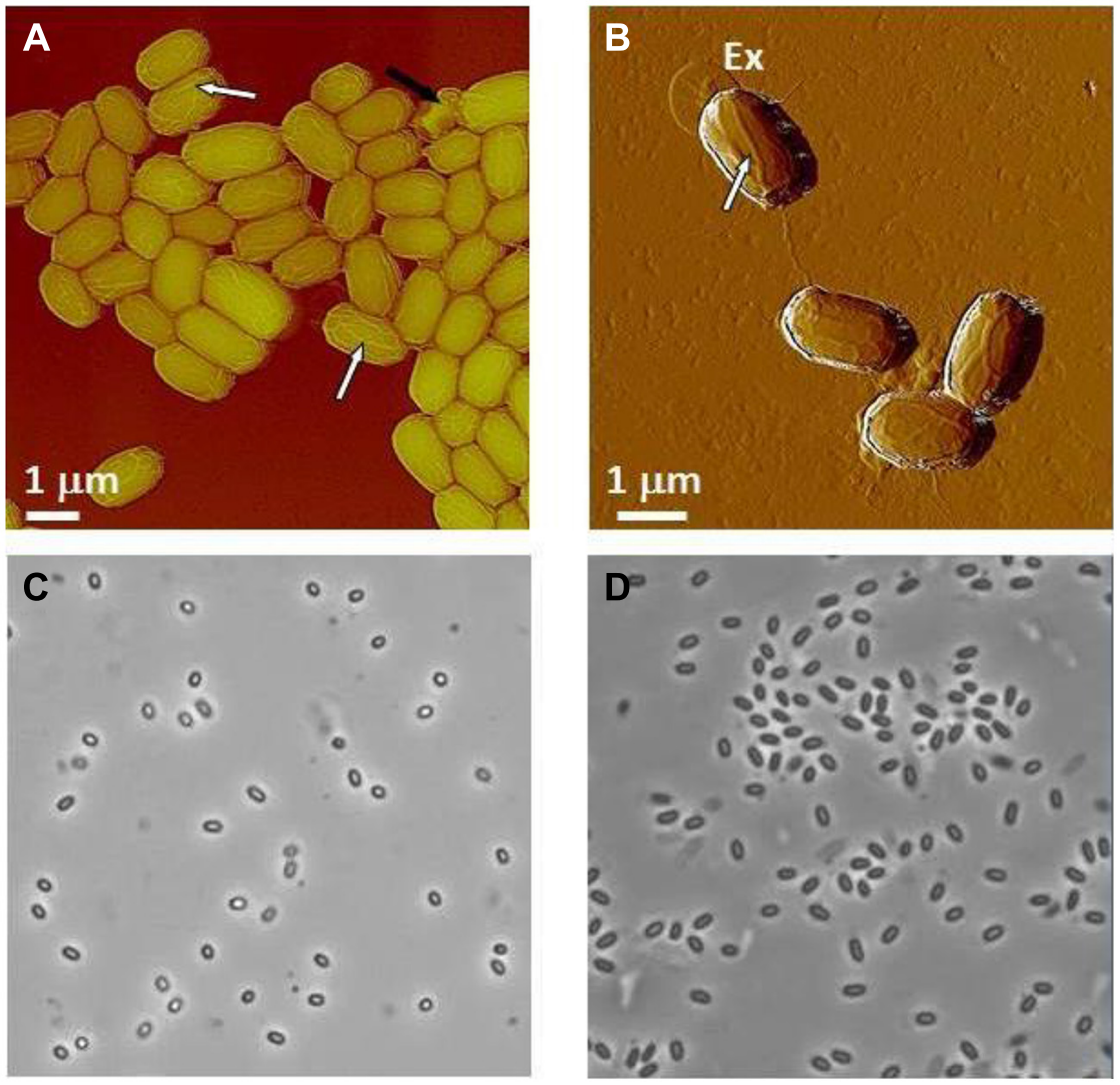
**Characterization of *Bacillus* spores.** **(A,B)** AFM images of native air-dried spores. **(A)** Height image of *B. anthracis* Sterne spores and **(B)** amplitude image of *B. thuringiensis* spores. In both images surface ridges extending along the entire length of spores (several surface ridges noted by white arrows) are seen. In **(A)** a collapsed spore is indicated with a black arrow. In **(B)** an exosporia is indicated with Ex. **(C,D)** Phase contrast microscopy images of *B. anthracis* Sterne spores **(C)** and *B. thuringiensis* spores **(D)**.

Typical AFM images of *B. anthracis* Sterne and *B. thuringiensis* spores are presented in **Figures 9A,B**, respectively. The corresponding optical phase microscopy is shown in (**Figures 9C,D**), demonstrating that these spores are phase bright (refractile, ungerminated). The vast majority of spores (**Figures 9A,B**) are intact with heights within ranges indicated in **Table 6** and exhibiting surface ridges (some indicated with white arrows) extending along the long axis of the spore. These ridges are the characteristic attribute of air-dried *Bacillus* spores (Plomp et al., 2005a,b,c, 2014) and appear due to coat folding caused by changes in spore size upon dehydration (Driks, 2003; Westphal et al., 2003; Plomp et al., 2005a). Occasionally, collapsed spores (as one indicated with a black arrow in **Figure 9A**) with heights in the range of 200–500 nm are observed in the spore preparations. This phenomenon could be attributable to partial germination, and subsequent partial collapse of the germinated spore upon air drying due to germination-induced internal structural changes.

##### 3.3.1.2. γ-Irradiated *Bacillus* spores

**FIGURE 10:**
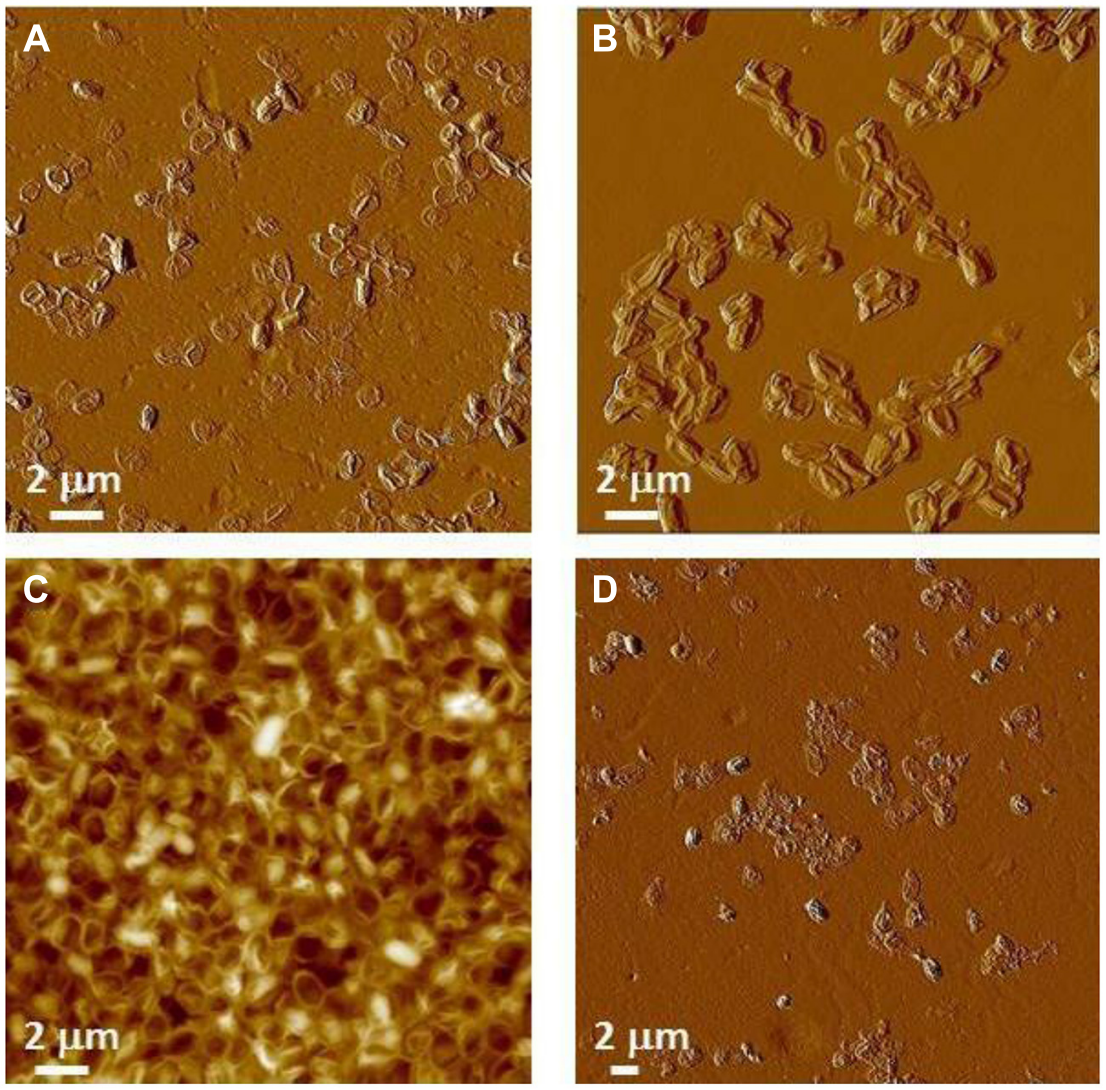
**Atomic force microscopy (AFM) images γ-irradiated (standard dose ≥ 2.5 × 10^6^ rads to assure sterility) air-dried *B. anthracis* Ames spores.** **(A)** Spores produced on Mueller Hinton-BBL; **(B)** spores produced on NSM agar; **(C)** spores produced on nutrient agar-BBL; and **(D)** spores produced spores produced on BHI-BBL.

**FIGURE 11:**
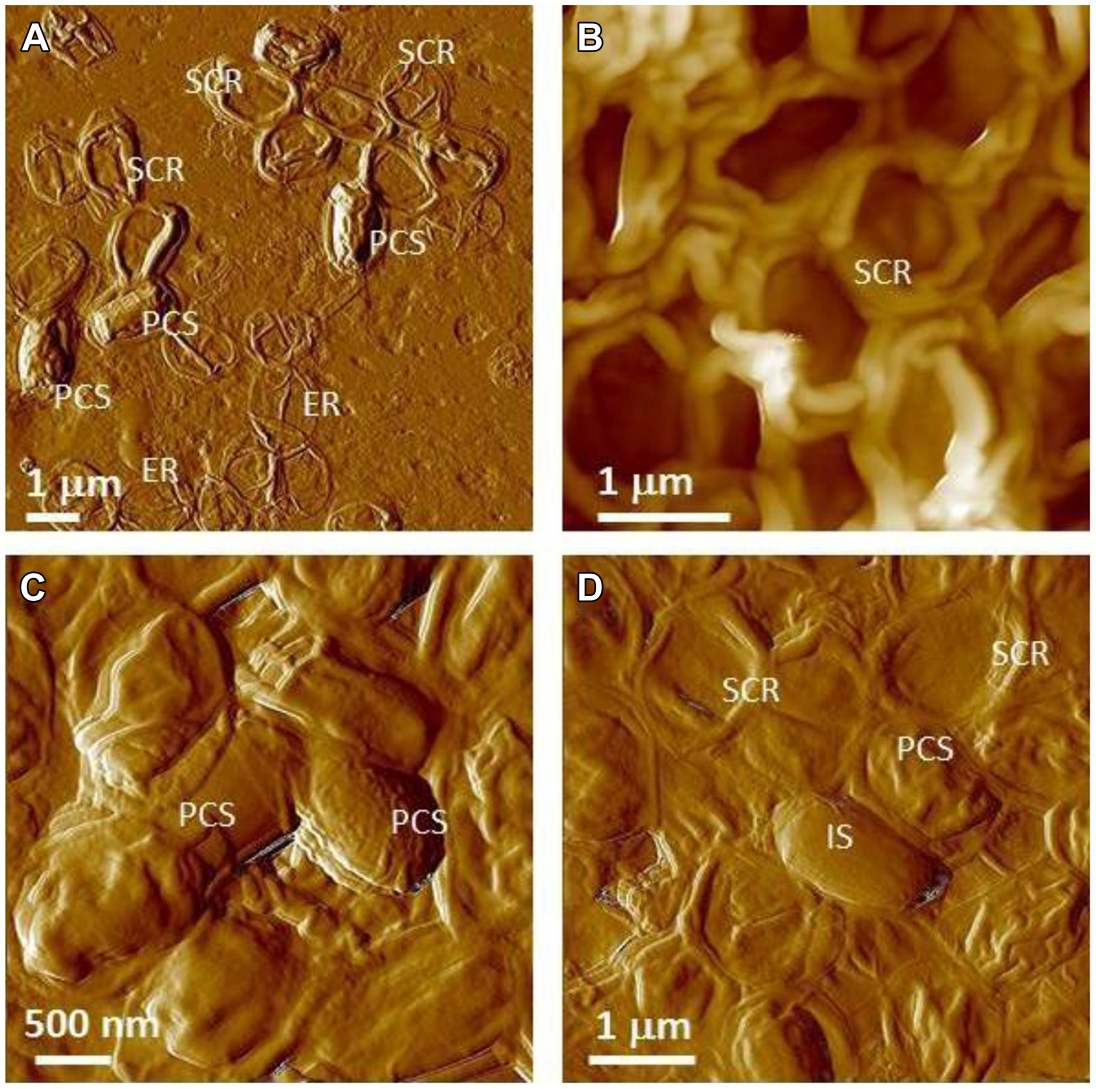
**Atomic force microscopy examination of γ-irradiated (≥2.5 × 10^6^ rads) *B. anthracis* Ames spore air-died samples.** **(A)** Spores produced on Mueller Hinton-BBL; **(B)** spores produced on NSM agar; **(C,D)** spores produced on nutrient agar-BBL. **(A–D)** are amplitude and height AFM images respectively.

Dauphin et al. (2008) reported that subjecting virulent *B. anthracis* spores at a concentration of 10^7^ CFU/ml to a dose of 2.5 × 10^6^ rads results in complete spore inactivation (sterilization). The spore images shown below in **Figures 10 and 11** were certified as sterilized for shipping and likely to have received a standard dose higher than 2.5 × 10^6^ rads to assure sterility. Specifically, we characterized γ-irradiated *B. anthracis* Ames spore samples produced on nutrient sporulation medium (NSM) agar, Nutrient agar-BBL, Mueller Hinton-BBL, and brain heart infusion (BHI)-BBL using AFM imaging and demonstrated that upon dehydration, as illustrated in **Figure 10**, the architecture of these spores collapsed. AFM examination of irradiated spores prepared as air-dried samples revealed further pronounced morphological and structural differences from native spores (**Figures 9A,B**). As illustrated in **Figure 11**, air-dried samples of γ-irradiated spores comprised partially collapsed spores (PCSs), spore coat remnants (SCRs), and exosporia remnants (ERs). The heights of PCS were in the range of 400–600 nm, which is significantly lower than the height of an air-dried non-irradiated native spores (**Table 6**). The thickness of SCR and ER was in the range of 100–250 and 40–45 nm respectively, which are comparable with the dimensions of native spore coats and exosporia. Only a small proportion of the air-dried samples of γ-irradiated *B. anthracis* Ames spores were present as intact spores (IS, **Figure 11D**) exhibiting heights equal to non-irradiated air-dried *B. anthracis* spores.

These studies demonstrated that exposure to sterilizing γ-irradiation produced profound structural changes in *B. anthracis* spores. Irradiation damages spore internal structural integrity (membranes, cortex etc.) and causes either partial (PCS) or complete (SCR) evacuation of the spore core. It is likely that in the hydrated sample, internal spore components that have sustained damage from the irradiation treatment (protein, DNA, ribosomes, small molecules, etc.) have partially/or completely diffused from the spore core into the bulk liquid phase. Note, that the leakage of spore core contents into the bulk media could also adversely affect biochemical and chemical analysis of the collected irradiated sample. Because of the significant internal structural damage induced by irradiation, the dehydration of irradiated spores suspended in liquid resulted in their collapse (**Figures 10** and **11**).

**FIGURE 12:**
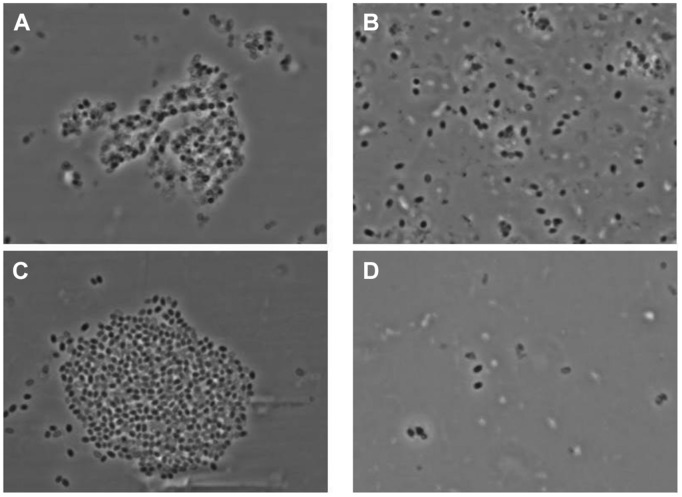
**Phase contrast microscopy images of γ-irradiated (≥2.5 × 10^6^ rads) *Bacillus anthracis* Ames spores **(A)** produced on Mueller Hinton-BBL, **(B)** produced on NSM agar, **(C)** produced on nutrient agar-BBL, and **(D)** produced BHI-BBL**.

There was an excellent cross-correlation between phase contrast optical microscopy and AFM in the characterization of the irradiated spore samples. Thus, as seen in **Figure 12**, phase contrast microscopy demonstrated that the vast majority of spores in irradiated samples were either phase dark, which corresponds to the evacuation of the spore coat, or spore ghosts, which corresponds to SCRs.

**FIGURE 13:**
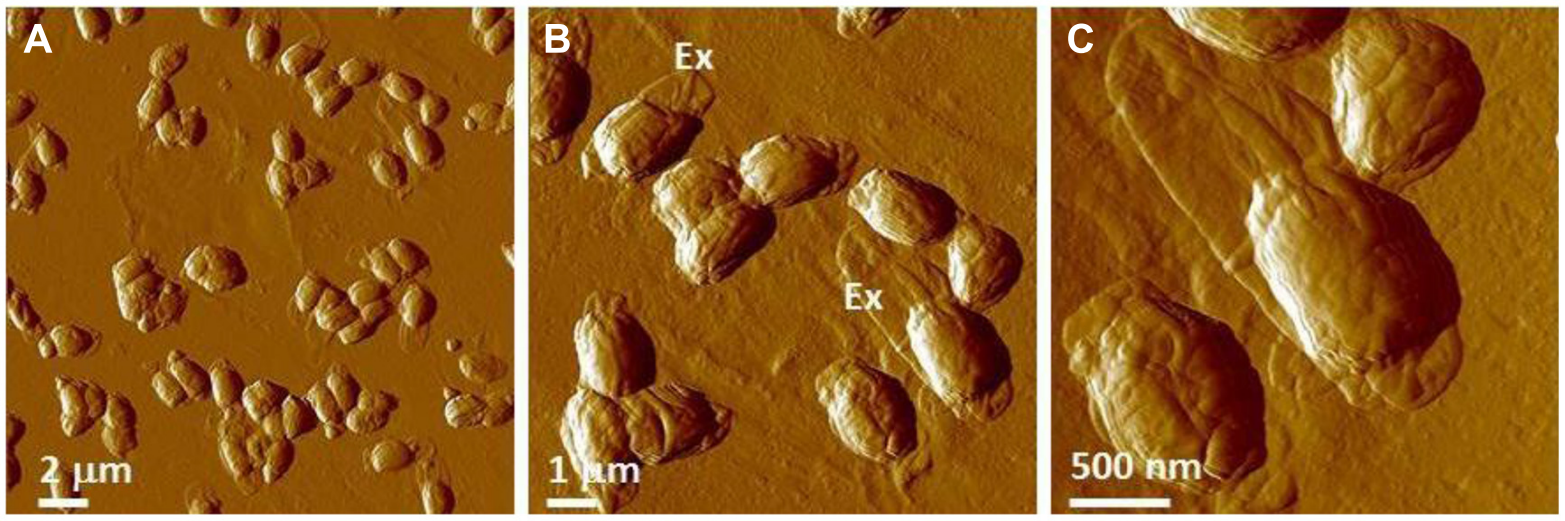
**AFM images of γ-irradiated (≥2.5 × 10^6^ rads) *B. thuringiensis* spores in (A) a low magnification AFM image.** In a higher magnification AFM image **(B)**, exosporia (denoted with Ex) are observed. Image **(C)** is a close-up image taken of the lower right area in panel **(B)**.

With AFM of air-dried spores for γ-irradiated *B. thuringiensis* spores, similar types of ultrastructural damage and collapse have been observed (**Figure 13**). These samples comprise PCS with heights in the range of 30–40% of the height of non-irradiated native air-dried *B. thuringiensis* spores (**Table 6**, Plomp et al., 2005a).

#### 3.3.2. AFM Characterization of Chlorine Dioxide-Treated *Bacillus* Spores

**FIGURE 14:**
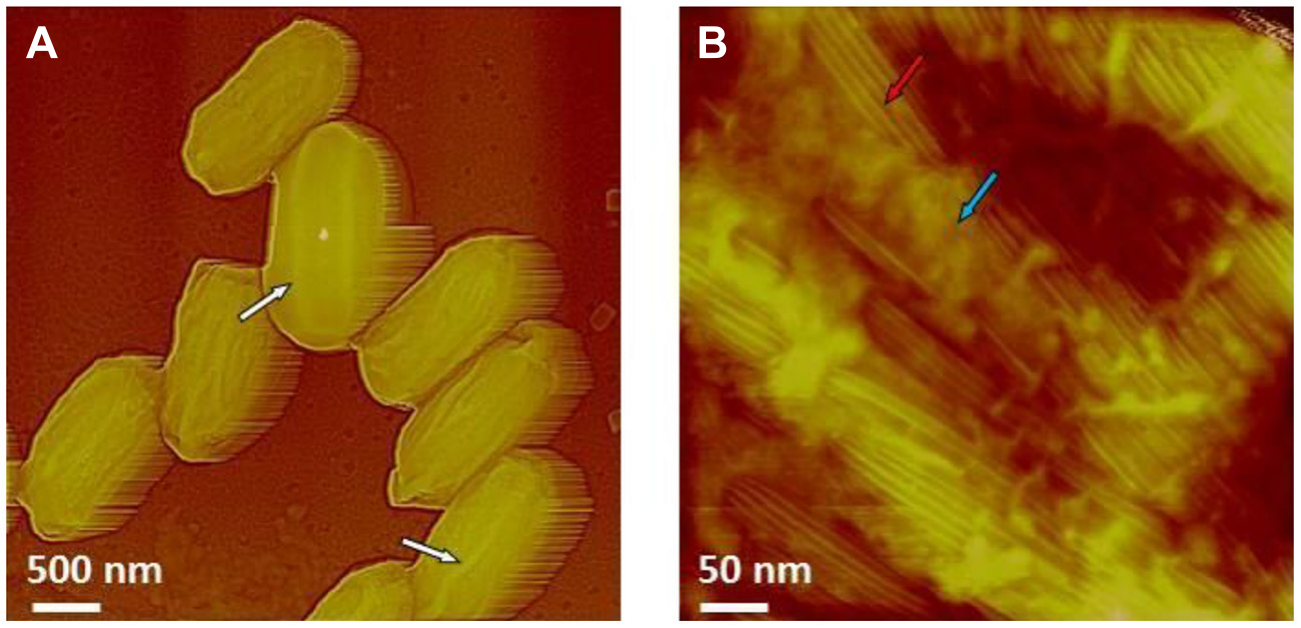
**(A,B)** AFM images of *B. atrophaeus* spores exposed to a sterilization (6-log kill) dose of ClO_2_ (500 ppm × 5 h = 2500 ppm-h). In **(A)** surface ridges extending along the entire length of spores (several surface ridges noted by white arrows) are seen. In **(B)** high-resolution AFM image showing the regular rodlet structure (red arrow) and patches of an amorphous outermost layer (blue arrow), both characteristic to native, air dried *B. atrophaeus* spores.

Chlorine dioxide is sporicidal and a well-known decontamination regime for spores of *B. anthracis*. As illustrated in **Figure 14**, *B. atrophaeus* spores exposed to sporicidal levels of chlorine dioxide (dose = 2500 ppm-h) remain intact upon air-drying. The height of the chlorine dioxide-treated spores varies in the range of 500–650 nm, which corresponds well with the height of native air-dried *B. atrophaeus* spores (Plomp et al., 2005a). Similarly, the morphology of ClO_2_-treated *B. atrophaeus* spores is indistinguishable from the morphology of untreated spores with pronounced surface ridges seen in **Figure 9A**. Furthermore, ClO_2_ is a selective oxidant affecting cysteine, tryptophan and tyrosine amino acids in proteins and is not expected to grossly alter spore coat ultrastructure. Indeed, the high-resolution spore coat architecture and topology of ClO_2_-treated spores is unaltered in character from those of native air-dried spores (Plomp et al., 2005a,b,c, 2014), with rodlet coat structures and patches of an amorphous outermost layer clearly seen (**Figure 14B**).

#### 3.3.3. AFM Characterization of Spore Responses in the Fully Hydrated vs. Air-Dried State

**FIGURE 15:**
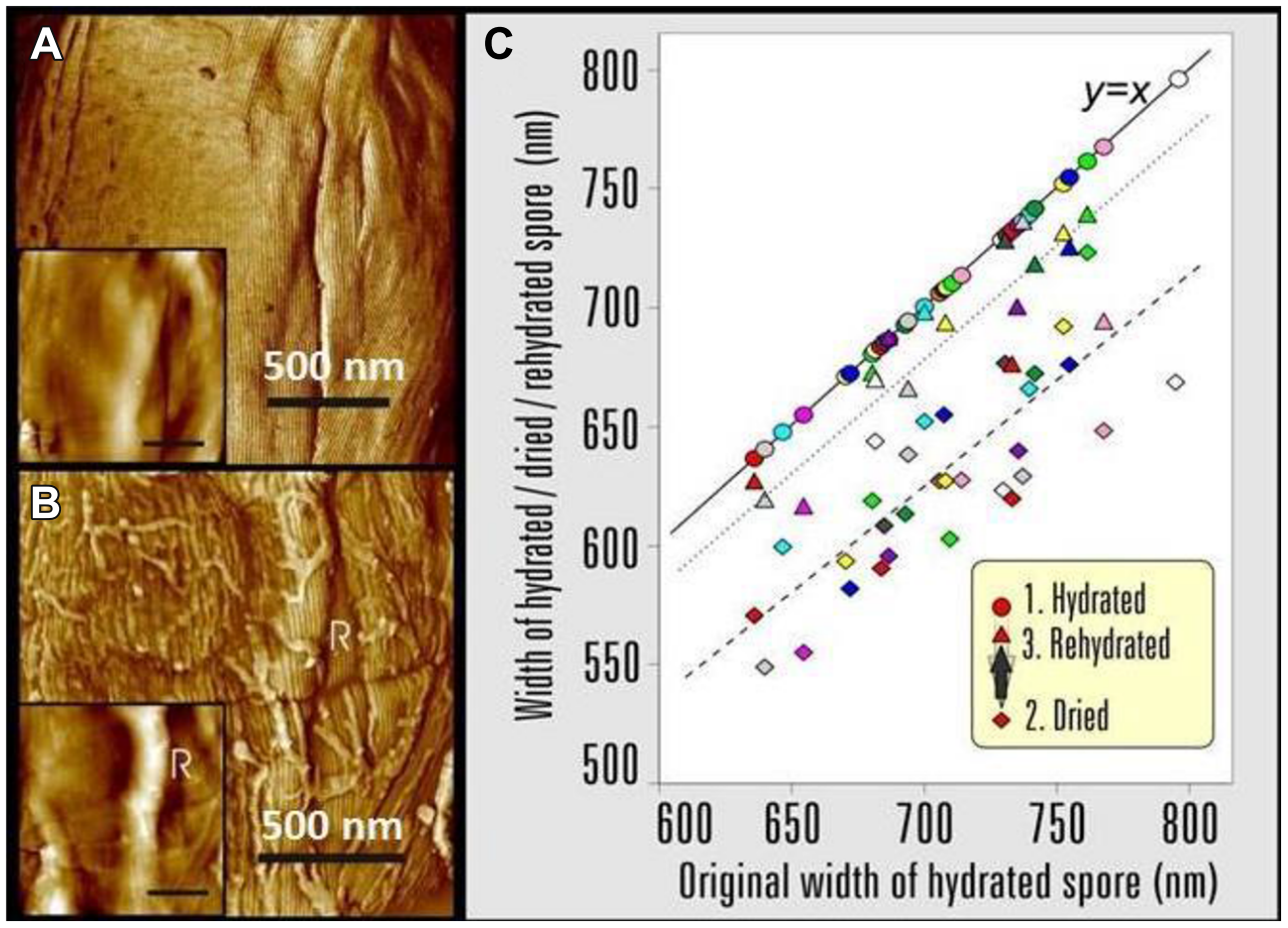
**AFM images showing the effects of changing the *B.atrophaeus* spore environment form hydrated to dehydrated states.** **(A)** Phase image and height image (inset) detail of a *B. atrophaeus* spore in water, showing rodlet spore coat structure and several shallow wrinkles. **(B)** The same spore after drying, showing rodlet structure (with many adsorbed stray rodlets, which sedimented from the bulk solution upon drying of the sample) and a 60-nm high ridge (indicated with R). The graph **(C)** shows spore width variations of 35 individual *B. atrophaeus* spores, as a function of the size of the originally hydrated spore, followed by dehydration (24 h; diamonds, dashed trend line), then rehydration (2 h; triangles, dotted trend line). For ease of comparison, the original hydrated spore width is (redundantly) depicted as circles, which by definition lie on the solid *y = x* line. Thus, the three data points for one individual spore, depicted with the same color, are all on the same vertical line. Several spores detached from a substrate during rehydration experiments resulting in a smaller amount of experimental rehydration points (triangles). On average, spore size is reduced to 88% for dried spores, and returns to 97% of the original width for rehydrated spores. Images reproduced, with permission from Plomp et al. (2005a), Copyright © (2005). The Biophysical Society. Published by Elsevier, Inc.

Atomic force microscopy allows a direct comparison of fully hydrated and air-dried native spores visualized under water and in air, respectively. Particularly, AFM studies of fully hydrated *B. atrophaeus* (Plomp et al., 2005a, 2007a), *B. anthracis* (Plomp and Malkin, unpublished results) and *Clostridium novyi* NT spores (Plomp et al., 2007b) demonstrated that high-resolution attributes (i.e., rodlet, honeycomb, and inner coat layer structures), maintained the same patterns, lattice periodicities, and step heights as seen with air-dried spores (Plomp et al., 2005a,b,c, 2007b, 2014; Elhadj et al., in preparation). Furthermore, the ability of the coat to fold and unfold concomitant with changes in spore size was suggested (Driks, 2003; Westphal et al., 2003) based on measurements of *B. thuringiensis* spore dimensions induced by humidity transients. Images of a fully hydrated *B. atrophaeus* spores are presented in **Figure 15A**. Surface ridges, the prominent structural features of air-dried spores (**Figure 9**), are typically absent from the surface of fully hydrated spores (**Figure 15A**). The direct visualization of 35 individual spores was performed to probe the dynamic response of their aqueous to aerial phase transition (Plomp et al., 2005a). Spores were visualized under water, then air-dried for ∼40 h and imaged in air (65% RH), then re-imaged after rehydration. Typical examples of hydration/dehydration ultrastructural transitions are presented in **Figures 15A,B**. As illustrated in **Figure 15A**, the coat of a fully hydrated spore appears to be tightly attached to the cortex and upon dehydration (**Figure 15B**) forms an ∼50 nm surface ridge/fold extending along the entire length of the spore.

The changes in spore surface architecture with dehydration were accompanied by a decrease in spore size. As illustrated in **Figure 15C**, the average width/height of 35 individual air-dried spores was reduced to 88% of the size measured for fully hydrated spores. The rehydration of air-dried spores, by placing them in water for 2 h, restored the spores to 97% of their original size, thereby establishing the reversibility of the size transition concurrent with rehydration (**Figure 15C**).

The observed decrease in the width of bacterial spores with dehydration is apparently due to the contraction of the spore core and/or cortex. The spore coat itself does not shrink/expand but is flexible enough to compensate for the internal volume decrease of core/cortex compartments by surface folding and the formation of ridges. These studies establish that dormant spores are dynamic physical structures, which exhibit profound morphological and structural responses with changes in its natural environment. These changes play important roles in selecting and implementing successful decontamination strategies.

### 3.4. Bio-indicators for the Inactivation of *Bacillus spp.* Spores by ClO_2_ Gas

As a gas-phase sporicide, ClO_2_ has advantages over other gas-phase sterilizing agents that leave residues or may pose health hazards (Bruch, 1973; Dolovich et al., 1984; Chapman et al., 1986; Lettman et al., 1986; Lykins et al., 1994; Muttamara et al., 1995). The efficacy of ClO_2_ decontamination treatments are typically verified through the use of spore BIs. We present extensive statistical analysis and modeling results that can be used to predict survival probabilities for three species of spores at varying doses of ClO_2_ by analyzing its effectiveness to kill 6 logs of bacterial spores (sterilize). Our analysis includes four lots of *B. atrophaeus* spore strips with different Decimal reduction (D) values, which are the times to reduce surviving spores by 90%, for ethylene oxide (D_EtO_) to allow their use as BI’s to monitor process efficacy in large-scale emergency decontamination efforts, as well as the spores of *G. stearothermophilus* and *B. thuringiensis*, a close relative of *B. anthracis*. Parameters contributing to the efficacy of spore sterilization by ClO_2_ include, but are not limited to, ClO_2_ concentration, RH, temperature, exposure time, and the BI packaging materials.

The data in **Figures 5A–D** clearly demonstrate that the probability, with a confidence level of 95%, of a spore surviving greatly diminishes as dose increases. In fact, the correlation between increasing [ClO_2_] and sporicidal activity is strong for all species and lots tested. At a constant spore strip population (1.2 × 10^6^), exposure time, and %RH (79%), the percent of spore strips killed increases (probability of a spore surviving decreases) as a function of increasing dose (dose ≡ ppm ClO_2_ × exposure time) as the gas concentration increases (**Figures 5A,B**). Similarly, we have also found that for a constant [ClO_2_], RH, and spore population, increasing the dose by increasing the exposure time also results in higher levels of kill (lower probability of survival, **Figures 5C,D**).

A particular point of interest is the dose at which the predicted spore survival probability is at most 10^-6^ (the EPA target) with 95% confidence. If we consider species *B. atrophaeus* as an example, for which there is a D_EtO_ value of 3.1 or 5.0, an RH of 0.79, and take the number of spores to be 10^6^ (not stratifying by packaging), then the dose at which a 6-log drop is achieved is, with 95% probability, between 268 and 592 or between 628 and 822 ppm-h, respectively (data not shown), which is significantly less than the dose of 2500 ppm-h required in some whole building decontaminations. This suggests that a lower D_EtO_ value correlates with a greater susceptibility to ClO_2_, a phenomenon that we also (see graphically in **Figures 6A–D**). Though *B. atrophaeus* spores are accepted as a standard test organism for sterility (Rosenblatt et al., 1987), the strains of *G. stearothermophilus* and *B. thuringiensis* spores assayed required higher ClO_2_ doses (at 0.79 RH), between 1235–2415 and 1497–2063 ppm-h, respectively, to achieve the same spore survival probability (≤10^-6^) with 95% confidence (**Figures 5A,B**).

With [ClO_2_] = 500 or 1000 ppm and exposure time fixed at 4 h, we varied the %RH (40–90%) of the exposure chamber to assess the bioeffects of humidified ClO_2_ on two lots of *B. atrophaeus* BI’s with D_EtO_ values of 3.3 and 3.8 (**Figures 6C,D**, respectively). We found, in general, that increasing %RH at a given dose increases the level of kill (**Figures 7A,B**). Additionally, to achieve equivalent levels of kill at a fixed ClO_2_ dose, higher RH is required for *B. atrophaeus* strips with a higher D_EtO_ value (**Figures 7A,B**). In general, we found that RH levels of ≥80% are optimal for ClO_2_ sporicidal bioeffects over a wide range of doses.

The humidity dependence of ClO_2_ sterilization may be twofold. Increasing RH results in an increase in localized sorption of water molecules on the spore surface, causing the spore to swell and hypothetically resulting in increased pore size through which ClO_2_ can pass (Westphal et al., 2003). ClO_2_ partitioning from the gas phase into aqueous solution is about 1:40, and it has been reported that ClO_2_, a radical, is a dissolved gas in solution (Aieta and Berg, 1986; Simpson et al., 1993). Therefore, as RH increases, thus does the local concentration of ClO_2_, and the spore becomes more swollen, perhaps more readily passing water and its associated dissolved ClO_2_ gas through opened channels. Other authors have reported a similar relationship between increased RH during ClO_2_ treatment with increased mortality of vegetative bacterial cells (Han et al., 1999, 2001).

We consistently observed no statistically significant difference in spore killing between BI’s packaged in 1059B medical grade Tyvek versus no packaging (**Figures 5–7**). Identical lots of *B. atrophaeus* log 6 spore strips, 1161841 and 1161911, with D_EtO_ values of 3.3 and 3.8, respectively, and packaged by the manufacturer in their standard and industry-accepted medical grade glassine, yielded grossly inconsistent results when exposed to a wide range of ClO_2_ doses (data not shown) – at 2000 ppm-h and 80% RH, 90% of the spore strips (lot 1161841) were killed when packaged in Tyvek and there was no effect on the strips packaged in glassine (**Figure 7C**). A much greater dose (∼20,000 ppm-h) was required to kill 100% of the *B. atrophaeus* strips packaged in glassine (**Figure 7D**). **Figure 7E** shows predicted probabilities of a spore remaining live after treatment, for Tyvek and no packaging, for all three species (two different D_EtO_ values for *B. atrophaeus*) and with RH = 0.79 and the number of spores fixed at *N* = 10^6^.

## 4. Bacterial Spore Properties – Mechanisms of Resistance and Killing

The novel ClO_2_ decontamination technologies mentioned above are laboratory inventions that were patented, commercialized, and adapted for actual field use in austere environments (e.g., far-forward military deployments or global humanitarian relief in third-world countries, etc.). The FDKs were adapted from the COTS version of NSRDEC’s decontamination technologies and became an important contributor for global public health organizations (MSF, WHO, Public Health Canada, NIH) in sterilizing Ebola-contaminated medical equipment and preventing the spread of disease at remote clinical sites in West Africa. All decontamination technologies have to be validated for efficacy in the laboratory and during their actual field use. Lethal chemical agents typically inactivate viruses by damaging the protein capsid or DNA (Kingsley et al., 2014). While the Ebola virus itself is an enveloped virus and relatively fragile, the virus is classified as Biosafety level 4 (BSL-4) due to the severity of disease it causes in humans, and presently there is no standard test assay for the Ebola virus under representative conditions even in a high-level containment facility. Bacterial spores, therefore, provide the standard assay for assuring sterility with deployed FDKs and other decontamination technologies, because of the extreme resistance of bacterial spores to chemical (and other) decontamination methods. Consonant thereto, we review bacterial spore properties, structures, and resistance mechanisms and focus on the mechanisms through which ClO_2_ inactivates bacterial spores as the indicators of efficient bio-decontamination.

### 4.1. Bacterial Spores – Background

Members of bacteria of *Bacillus* and *Clostridium* species and their close relatives can form metabolically dormant spores, generally when the environment no longer can support growth (Setlow, 2006; Leggett et al., 2012; Setlow and Johnson, 2012). Spores are extremely resistant to all manner of harmful treatments, and can remain dormant for years. However, if given the proper stimuli, generally nutrients such as amino acids or sugars, spores can return to life in minutes and outgrow into vegetative cells (Setlow, 2013). During subsequent growth, the growing or stationary phase cells of some spore-formers can secrete toxins or deleterious enzymes, and these agents can cause food spoilage or food poisoning and other human or animal diseases. As a consequence, there is much interest in the mechanisms of spore resistance to and the killing of spores by different treatments including high pressure, radiation, chemicals, plasma, or heat. One factor that is often overlooked in thinking about these mechanisms is the significant heterogeneity in properties of individual spores in genetically homogeneous spore populations, and this is seen in levels of spore resistance and in rates of spore germination (Setlow et al., 2012). The reasons for some of this heterogeneity are known, in particular some of the causes of the variability in rates of germination between individual spores. However, the reasons for the variability in resistance properties between individual spores in populations are not clear.

### 4.2. Spore Structure, Components, Properties, and their Role(s) in Spore Resistance

**FIGURE 16:**
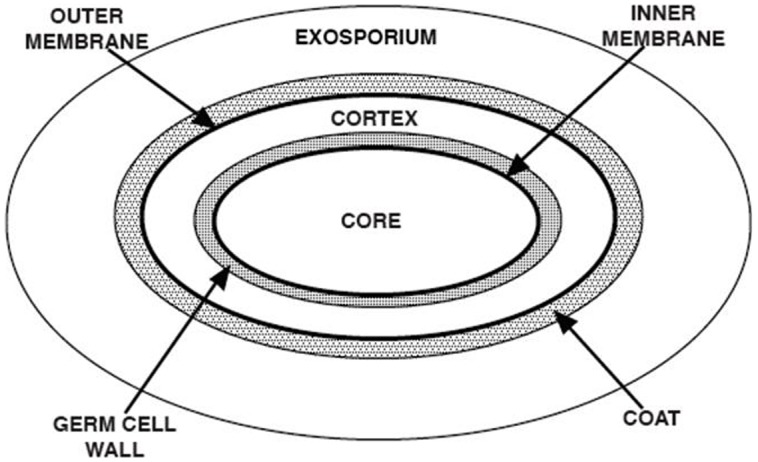
**Spore structure.** The various layers of a typical spore are shown in schematic form, and the various spore layers are not drawn to scale. Note that the exosporium is not present in spores of all species.

A number of novel spore structures and many spore-specific components and features are crucial for imparting various spore resistance properties (**Table 7**). Spores of all species appear to have a generally similar basic structure (**Figure 16**), with the exception of the outermost exosporium layer that is present in spores of some species, but not all (Gerhardt, 1967; Henriques and Moran, 2007; Driks, 2009; McKenney et al., 2013). The large balloon-like exosporium is composed of proteins, sugars, and lipid, and it is present in many spore formers that can cause disease, including the members of the *Bacillus cereus* group and *Clostridium difficile*, but is absent from the model spore former *B. subtilis*. This structure appears to be important in spore adhesion properties, and in restricting access of antibodies to the spore coat layer below the exosporium, but plays no major role in most spore resistance properties (Setlow, 2006; Henriques and Moran, 2007). The exosporium may also play some role in virulence of spores, but this role is not yet clear.

**Table 7:** Mechanisms of spore killing by and resistance to various agents^∗^.

Type of agent	Mechanisms of killing	Mechanisms of resistance
HPP	Probably protein damage	Low core water content
UV, γ-radiation, nitrite, formaldehyde	DNA damage	α/β-type SASP, DNA repair, and perhaps IM impermeability
Oxidizing agents (OCl^-^, ClO_2_, O_3_)	IM damage	Spore coat/outer membrane
H_2_O_2_	Probably core protein damage	α/β-type SASP, low core water
OH^-^, wet heat, some oxidizing agents	Inability to germinate	Spore coat/outer membrane
Strong mineral acid	Explosive rupture of IM	Not studied
Plasma	Protein or DNA damage^1^	α/β-type SASP, spore coat, DNA repair
Wet heat	Protein damage	α/β-type SASP, low core water content
Dry heat	DNA damage	α/β-type SASP, DPA, DNA repair

*^∗^Data for these conclusions are from Gerhardt and Marquis (1989), Setlow (1994, 2001,2006; 2007a), Coleman et al. (2007, 2010), Leggett et al. (2012), Setlow and Johnson (2012), Reineke et al. (2013a,b), Moeller et al. (2014), Setlow et al. (2014)*.

*^1^Plasma with UV-C kills spores by DNA damage, while UV-C free plasma likely kills by protein damage*.

Moving from the exterior inward, the spore coat is found under the exosporium, and is composed of many spore-specific proteins assembled in a number of layers (Henriques and Moran, 2007; McKenney et al., 2013). The coat can contain up to 50% of total spore proteins, and protects more inner spore layers such as peptidoglycan from attack by lytic enzymes such as lysozyme, and also by lytic enzymes of predatory eukaryotes. The coat is also probably crucial in the resistance of spores to a variety of chemicals, including chlorine dioxide (ClO_2_) and sodium hypochlorite (bleach, OCl^-^), that are widely used for spore decontamination. As a consequence, spores that have defective coats because of mutation(s) or chemical de-coating are sensitized to these oxyhalogens and to many other types of chemicals, although not to all. Indeed, the coat plays only a minor role in spore resistance to chemicals such as H_2_O_2_, nitrous acid, acid and alkali, and DNA alkylating agents (**Table 7**). In a few cases, enzymes present in spore coats, superoxide dismutases (SODs catalyze the dismutation of superoxide, O_2_^-^, to O_2_ or to H_2_O_2_) and catalases (catalyze the disproportionation of H_2_O_2_ to O_2_ and H_2_O), have been shown to play roles in spore resistance to some oxidizing agents, both *in vitro* and in *in vivo*-like environments (Henriques and Moran, 2007; Cybulski et al., 2009; McKenney et al., 2013).

Beneath the spore coat is found the outer membrane, which is a critical element in spore formation. While it is possible that the outer membrane is a permeability barrier in dormant spores (Gerhardt and Black, 1961; Rode et al., 1962), this role is not clear, and there are no mutants that specifically affect the spore’s outer membrane. The outer membrane is also lost either partially or completely in spores with defective coats, and the outer membrane is also removed by chemical de-coating treatments (Buchanan and Neyman, 1986). In general, the outer membrane specifically is thought not to play a major role in spore resistance properties, but it is actually difficult to separate the roles of the outer membrane and coat in determining spore resistance properties.

Under the outer membrane are two layers of peptidoglycan (PG); first, the large spore cortex, then the thinner germ cell wall that comprises the minority of total spore PG (Popham, 2002). The germ cell wall PG has a structure that appears identical to that of growing cell wall PG structure, and cortex PG has a structure similar to that of the germ cell wall PG, but with several cortex-specific modifications. As a consequence, in the process of spore germination, cortex-lytic enzymes (CLEs) degrade cortex PG, while leaving the germ cell wall intact and allowing this structure to form the cell wall of the outgrowing spore (Setlow, 2013). The cortex’s main role in spore resistance appears to be to maintain and possibly help establish the extremely low water content in the central spore core (25-50% of wet wt) that is crucial in spore resistance to wet heat (**Table 7**) and probably in spore dormancy (see below). There are a number of *B. subtilis* mutants that affect cortex PG structure. Some changes in cortex PG are associated with changes in spore wet heat resistance, and, in some cases, changes in cortex PG are associated with changes in spore core water content (Zhang et al., 2012).

Under the germ cell wall is found the second spore membrane called the inner membrane (IM). This IM has a phospholipid and fatty acid composition similar to the plasma membrane of the growing or sporulating cell (Griffiths and Setlow, 2009). However, lipid probes in the IM are immobile, suggesting the IM is not as fluid as membranes in growing cells, although IM fluidity is restored when spores complete germination and the core expands (Cowan et al., 2004). This expansion takes place in the absence of any ATP synthesis as the volume enclosed by the IM increases more than twofold. As with IM lipid mobility, IM permeability also rises dramatically, when the IM enclosed volume increases during spore germination. The low permeability of the dormant spore IM appears to contribute significantly to spore resistance to hydrophilic chemicals, as changes in dormant spore IM permeability due to changes in sporulation conditions, primarily temperature, parallel changes in IM permeability (Cortezzo and Setlow, 2004). It has been suggested that the IM is held in some sort of compressed state in the dormant spore, and this suggested structure is consistent with the extremely low permeability of the IM observed in dormant spores, even to a hydrophobic molecule such as methylamine, and perhaps even to water (Setlow and Setlow, 1980; Swerdlow et al., 1981; Cortezzo and Setlow, 2004; Sunde et al., 2009; Ghosal et al., 2010; Kaieda et al., 2013; Kong et al., 2013). Unfortunately, the precise structure of dormant spores’ IM and how this structure influences IM permeability are still not clear. This is important information, because a number of key proteins in spore germination are in the IM (Setlow and Johnson, 2012; Setlow, 2013), and IM properties will most likely influence the function of these germination proteins.

The final spore layer is the central core, the site of spore DNA, RNA, and most spore enzymes. As noted above, the core has low water content, and this undoubtedly is the reason that soluble, mobile and active proteins in growing cells are immobile and inactive in the spore core (Gerhardt and Marquis, 1989; Setlow, 1994; Cowan et al., 2003). Indeed, while the dormant spore core has enzymes such as catalases and superoxide dismutases that play major roles in the resistance of growing cells to oxidizing agents, these enzymes play no role in dormant spore resistance, presumptively because they are inactive in the environment of the dormant spore core (Setlow, 2006; Leggett et al., 2012). The low core water content is most likely the major factor in spore resistance to wet heat (Gerhardt and Marquis, 1989). There are also at least three other unique spore core features to consider. First, the core pH is ∼1.5 units lower than the pH in a growing cell or in the mother cell compartment of the sporulating cell (Setlow and Setlow, 1980). The importance of this low core pH in spore resistance is not clear, since the core pH can be elevated 1.5 units with no effects on spore dormancy or resistance (Swerdlow et al., 1981). However, the decrease in spore core pH during sporulation appears to be important in the modulation of enzyme activity in the developing spore late in sporulation (Setlow, 1994). Enzymes that are modulated in this way include the zymogen of the GPR protease that auto-activates at a low pH, and phosphoglycerate mutase [catalyzes the interconversion of 3-phosphoglycerate (3-PGA) to 2-phosphoglycerate] that becomes much less active at pH ∼6.3, thus causing the accumulation of a large amount of 3-PGA in the dormant spore (Illades-Aguiar and Setlow, 1994; Setlow, 1994). This 3-PGA depot is rapidly catabolized to generate ATP in the first minutes of spore germination, during which core pH rises to ∼7.8 and core water content rises to ∼80% of wet wt (Setlow and Johnson, 2012; Setlow, 2013).

A second unique feature of the spore core is the high level (∼20% of core dry wt) of pyridine-2,6-dicarboxylic acid [dipicolinic acid (DPA)], which exists as a 1:1 chelate with divalent cations, predominantly Ca^2+^ (CaDPA; Gerhardt and Marquis, 1989; Setlow, 2006; Setlow and Johnson, 2012). DPA is made in the mother cell compartment of the sporulating cell, and the accumulation of CaDPA in the core late in sporulation plays a significant role in reducing spore core water content (Paidhungat et al., 2000). CaDPA in the core also has significant affects on spore DNA photochemistry, and thus CaDPA accumulation in the core alters spore resistance to UV radiation, but actually sensitizes DNA to UV radiation at some wavelengths (Setlow, 2001, 2007b). While the spores’ huge CaDPA depot is stable for long periods in spores suspended in water even at relatively high temperatures (75–80 °C for *B. subtilis* spores), the spores’ entire CaDPA depot is released rapidly in the first minutes of spore germination (Setlow, 2013). In addition, treatment with a number of oxidizing agents alters spores in some fashion such that their CaDPA depot is released completely even at 75°C (Cortezzo et al., 2004).

Another notable unique feature of the spore core is the saturation of spore DNA with a group of novel small DNA-binding proteins, termed Small, Acid-Soluble Proteins (SASP) of the α/β-type, so named because the two major proteins of this type in *B. subtilis* were initially termed SASP-α and SASP-β (Setlow, 2001, 2007b). The amino acid sequences of these ∼60–75 residue proteins are unique and extremely well-conserved both within and across spore forming species, including members of both *Clostridiales* and *Bacillales*. The α/β-type SASP play major roles in protecting spore DNA against UV radiation (∼260 nm) by changing DNA structure from the B-form to an A-like form in which the UV photoproducts formed in growing cell DNA, including cyclobutane pyrimidine dimers and 6–4 photoproducts between adjacent pyrimidines, are not generated, and some of the latter UV lesions are extremely mutagenic and thus potentially lethal. In contrast, with α/β-type SASP saturated DNA the major UV photoproduct generated by ∼ 260 nm radiation is a novel thymine-thymine adduct initially called the spore photoproduct (SP), and spores have a number of enzymes that repair SP in a relatively error-free manner. Thus α/β-type SASP are a major factor in spore resistance to UV radiation and also to γ-radiation, although repair of DNA damage in spore outgrowth is also important (Setlow, 2001, 2007b; Moeller et al., 2014). The α/β-type SASP also protect spore DNA against other types of damage, particularly depurination by wet and dry heat, and also against a number of genotoxic chemicals, including nitrous acid and formaldehyde (Setlow, 2006). The structure of a complex of an α/β-type SASP bound to a short DNA fragment in conjunction with modeling studies based on this structure have elucidated the causes of these effects of α/β-type SASP on DNA properties at the atomic level (Lee et al., 2008).

The α/β-type SASP are degraded in the first minutes of spore germination and outgrowth (Setlow, 1994, 2013) in a process initiated by an endoprotease termed GPR that is specific for a conserved sequence found in all α/β-type SASP. Oligopeptides generated by GPR cleavage are then degraded rapidly to free amino acids that are re-utilized by the outgrowing spore for metabolism and protein synthesis. Spores in which α/β-type SASP are not degraded rapidly following spore germination, either because GPR is inactive or because an α/β-type SASP binds too tightly to DNA, exhibit slow outgrowth and in some cases decreased viability (Setlow, 1994; Hayes and Setlow, 2001).

### 4.3. Mechanisms of Spore Killing

Work primarily with *B. subtilis* spores, and some with spores of other species (Setlow, 2006; Setlow et al., 2014), has identified five different mechanisms for killing of spores by various agents (**Table 7**). These are: (1) DNA damage; (2) damage to the spores’ IM; (3) damage to some essential spore core protein; (4) explosive rupture of the dormant spores’ IM; and (5) destruction of one or more spore components that are essential for spore germination. It is important to note that spores apparently inactivated by the last mechanism may not actually be dead, but just cannot return to active growth, since spore germination is blocked. There are a number of examples of spores that appear dead because they can’t germinate, but return to life, when assisted in spore germination, generally by provision with a lytic enzyme that can degrade spores’ PG cortex (Setlow et al., 2002; Paredes-Sabja et al., 2009a,b; Burns et al., 2010). This has been observed with spores that are apparently inactivated by treatment with chemical agents that either remove and/or inactivate enzymes in spores’ outer layers such as CLEs and/or proteases that can activate CLE zymogens in spores of *Clostridium* species. Thus, it is crucial to ensure that a spore killing regimen has not simply rendered spores incapable of germinating, such that the treated spores cannot be revived by artificial germination treatments; a number of tests have been used to demonstrate this (Setlow et al., 2014).

Among the other four methods of spore killing, the rarest is probably explosive rupture of the dormant spores’ outer layers. This phenomenon has only been seen with spores incubated in rather high concentrations of strong mineral acids such as HCl or HNO_3_ (Setlow et al., 2002; Setlow, 2006). As would be expected, UV and γ-radiation kill spores largely by DNA damage, since loss of DNA repair capacity greatly decreases spore resistance to these treatments, and survivors of these radiation treatments also accumulate high levels of mutations. A number of genotoxic chemicals, including nitrous acid and formaldehyde, also kill spores by DNA damage, as does dry heat, which generates significant levels of abasic sites in DNA. However, the potentially genotoxic agent, H_2_O_2_, does not kill spores by DNA damage, because of the strong protection of DNA against H_2_O_2_ damage by the α/β-type SASP.

While the mechanisms of spore killing and of spore resistance are known for many of the agents used to kill spores in applied settings – wet heat, radiation, chemicals such as hypochlorite or chlorine dioxide - there is a lack of definitive studies on the exact mechanisms of spore killing by and resistance to high hydrostatic pressure (HP) and various types of plasmas. In the case of HP, treatments at HP ≈400–700MPa (levels proposed for food processing) do not efficiently kill spores, unless accompanied by elevated temperature (*T* = 90–121°C). Spore killing by HP treatments takes place in (at least) two steps; first, spores are germinated by the HP, then the much less resistant germinated spores are killed at the elevated temperatures at which HP treatments are carried out.

Generally, moderate HPs (100–350 MPa, *T* = 37°C) trigger germination by activating the same GRs that trigger spore germination with nutrients, while HPs of 400–900 MPa (*T* ≥ 50°C) appear to directly open the channel through which CaDPA is normally released during germination (Setlow, 2007a; Reineke et al., 2013a,b). Recent studies have indicated that HP treatments in the regime *P* = 550–692 MPa and *T* = 75–112°C induce spore activation (potentiate spores for germination), and this can even increase apparent spore titers (Luu et al., 2015). This latter effect has been seen with spores of the model spore former, *B. subtilis*, and also with spores of *B. amyloliquefaciens*, a suggested surrogate for *Clostridium botulinum* spores in HP studies (Margosch et al., 2004; Sevenich et al., 2014). Other recent work (Kong et al., 2014) with *B. subtilis* spores has shown that: (i) 140 MPa HP treatments as short as 10 s can commit spores to germinate for up to 10 min during holding at atmospheric pressure; and (ii) almost all spores go on to germinate over >45 min after a 10 s 140 MPa HP treatment during holding at 1 MPa, a pressure that alone does not induce germination. These latter findings indicate that at least some of the effects of 140 MPa of HP that can lead to spore germination are reversible, and thus the reversible events take place before events that irreversibly commit spores to germinate. It will be of interest to examine these same phenomena in spores exposed to HPs ≥500 MPa (the HP that opens channels for CaDPA release in *B. subtilis* spores).

In the case of cold plasma, when significant levels of UV photons are associated with a particular type of plasma, there is evidence that these plasmas kill spores by DNA damage and α/β-type SASP and DNA repair are important in spore resistance to such plasmas (Roth et al., 2010; Yardimci and Setlow, 2010; Klämpfl et al., 2012; van Bokhorst-van de Veen et al., 2015). For plasmas that have minimal associated UV photons, mechanisms of spore killing and resistance are less clear. There is certainly evidence that plasmas with oxygen in the feed gas can cause significant physical damage to spores by etching the spores’ outer layers. However, it is likely that this physical damage is not what actually kills spores, and research is needed to establish definitively mechanisms of spore killing by and resistance to various plasmas, including those that contain minimal levels of UV photons.

Surprisingly, wet heat treatment does not kill spores by DNA damage, even though the temperatures used for spore killing, ≥90°C, might be expected to generate many abasic sites in DNA due to depurination. However, α/β-type SASP protect DNA so well-against damage by wet heat that wet heat almost certainly kills spores by damage to one or more essential core proteins (Setlow, 2006; Coleman et al., 2007, 2010; Leggett et al., 2012). As might be expected, spores lacking the majority of their α/β-type SASP, termed α^-^β^-^ spores, are more sensitive to wet heat, which kills α^-^β^-^ spores by DNA damage including depurination. Some peroxides (e.g., H_2_O_2_), also kill α^-^β^-^ spores by DNA damage, although they probably kill wild-type spores by damage to some core protein(s).

While some toxic chemicals kill spores either by DNA damage or damage to some spore core protein(s), many toxic chemicals used in the killing of spores, including chlorine dioxide (ClO_2_), hypochlorite (OCl^-^), and iodine, kill spores by damaging the spores’ IM, such that the IM ruptures when the treated spores germinate (Cortezzo et al., 2004; Setlow, 2006; Leggett et al., 2012). Treatment with these agent(s) also seems to somehow alter the IM in dormant spores, such that: (i) the IM permeability increases dramatically; and (ii) the IM is less able to withstand a thermal stress, and DPA is released at much lower temperatures than corresponding untreated spores. Further indications of damage to spores by a number of oxidizing agents are that: (i) treated spores germinate quite slowly, even if some of the germinated spores do ultimately give rise to colonies upon plating; and (ii) recovery of colonies from such treated spores is extremely sensitive to increased salt concentrations in the recovery media. However, the nature of the damage affecting the spores’ IM leading to these effects is not known.

### 4.4. Summary

The AFM methods discussed previously provide information complementary to the spore inactivation mechanism discussion above by visualizing and characterizing spore morphological, dimensional, and coat structural attributes, particularly in response to sterilization and/or decontamination treatments by gamma-irradiation or ClO_2_. For example, irradiation treatments induced pronounced morphological and structural differences compared with native spores, including loss of refractility, decreased size, and damage to spore internal structural integrity, which is consistent with, but not necessary revealing of, DNA damage, the primary mechanism of bacterial spore inactivation by γ-irradiation. It is also possible that the sterilization treatment by γ-irradiation is extensive and induces further structural damage to already-killed spores as additional post-mortem events. Interestingly, ClO_2_-treated spores showed similar height, morphology, and high-resolution spore coat architecture and topology compared to native, untreated spores. Such observations with AFM are consistent with the known mechanisms of spore inactivation by ClO_2_ by acting on the IM as mentioned above, such that ClO_2_-killed spores treatments retain refractility (phase-bright) and size, but falter during germination and are unable to grow out (Young and Setlow, 2003).

Atomic force microscopy measurements also showed the reversibility of a size transition in fully hydrated, dehydrated, and re-hydrated environments. AFM characterization also showed that spore size decreases in dehydrated environments (air versus water), most likely due to contraction of the spore core and/or cortex. In contradistinction, the spore coat is more intractable, not shrinking or expanding with dehydration or hydration, respectively, but providing some flexibility to accommodate internal volume changes of spores by surface folding and the formation of ridges in air-dried spores that are absent from the surface of fully hydrated spores. Such results are complementary to and consistent with the development of spore BIs mentioned above. The spore BIs tested a number of strains, doses (ClO_2_ concentration × time profiles) and humid environments and found that %RH ≥ 70 was needed presumptively to promote spore swelling and facilitate internalization of ClO_2_ molecules to effectuate spore inactivation and oxidizing proteins associated with the spore IM. It is absolutely essential for the sterilization of Ebola contaminated devices and equipment in that the spore BIs provide a full and reliable assay of sterilization, to prevent the spread of disease, protect international healthcare workers on the front lines of global public health crises, and to care for the aﬄicted and save lives. Thus, the complementary information provided through bacterial spore mechanistic studies, AFM morphological and structural characterization, and spore BI are needed to provide the full assurance that NSRDEC’s novel decontamination technologies, to include the FDKs that operate at 70–90 %RH and >7000 ppm ClO_2_, are achieving sterilization when deployed in global crises, and the results reported above provide science-based solutions that satisfy this mission with confidence.

### 4.5. Conclusion

Moving exciting research findings from the benchtop to the hands of the end-user as novel technologies is a paramount goal of Science and Technology (S&T) for many government research and development (R&D) facilities. The novel ClO_2_ decontamination technologies invented at NSRDEC serve as a model for successful federal research and development work, Technology Transfer, commercialization, and deployment for actual use in the field. In fact, this work began with basic research in the mechanisms of bacterial spore inactivation, then transitioned to early exploratory research and development work focused on the chemical reaction kinetics and mechanisms of ClO_2_ formation occurring through a unique chemical effector. Later development work consisted of inventions, validating efficacy, obtaining patents, and eventually transitioning to private industry through Technology Transfer for commercialization in the marketplace. This technology has become a COTS item that enables its recent culmination as FDKs deployed at clinical sites in West Africa for the sterilization of Ebola-contaminated medical devices at a time when concern of the Ebola crisis in those regions and the fears of the virus spreading internationally were at their highest.

Scientists at NIH/NIAID and USAMRIID (The U.S. Army Medical Research Institute of Infectious Diseases) adapted the COTS version of NSRDECs novel decontamination technologies and developed compact, lightweight, easy-to-carry FDKs for use by global public health organizations such as MSF, the WHO, Public Health Canada, NIH, and the U.S. government to improve hygienic conditions in remote clinics where little infrastructure existed by sterilizing medical equipment and help fight the spread of a deadly disease that cost thousands of lives and untold costs in medical expenses caring for the aﬄicted.

Natick Soldier RD&E Center’s novel decontamination technologies were readily available to help others meet and overcome the challenges and concerns of this international public health through R&D that strives for such preparedness. These novel technologies decontaminate microbes in myriad applications, such as rinses, sprays, and gas for fresh produce; food contact and handling surfaces; PPE; textiles used in clothing, uniforms, tents, and shelters; graywater; airplanes; surgical instruments; and hard surfaces in latrines, laundries, and deployable medical facilities.

One strength of NSRDECs novel decontamination technologies is that the mechanisms of bacterial spore inactivation by ClO_2_ and the use of spore bio-indicators for ClO_2_ in decontamination procedures have been investigated thoroughly at University of Connecticut Health Center, Lawrence Livermore National Laboratories, Children’s Hospital of Oakland Research Institute, Stonehill College, and Brandeis University, making the fundamental science of ClO_2_ generation and decontamination well-understood, and NSRDECs technologies more assured and reliable. We have also reviewed mechanisms of bacterial spore inactivation by novel, emerging, and established non-thermal technologies for food preservation, such as HPP, irradiation, cold plasma, and chemical sanitizers, using an array of *B. subtilis* mutants to probe mechanisms of spore germination and inactivation, demonstrating that understanding the basic science allows the development of innovative technologies that can find solutions in diverse applications outside of the pasteurization and sterilization of foodstuffs.

## Conflict of Interest Statement

Christopher J. Doona, Florence E. Feeherry and Kenneth Kustin, as inventors from the U.S. Army Natick Soldier RD&E Center, receive royalties for patented technologies licensed to ClorDiSys Solutions, Inc. and used in the Field Decontamination Kits. The other authors declare that the research was conducted in the absence of any commercial or financial relationships that could be construed as a potential conflict of interest.
